# Evaluation of the antidermatophytic activity of potassium salts of *N*-acylhydrazinecarbodithioates and their aminotriazole-thione derivatives

**DOI:** 10.1038/s41598-024-54025-9

**Published:** 2024-02-12

**Authors:** Anita Ciesielska, Aleksandra Kowalczyk, Agata Paneth, Paweł Stączek

**Affiliations:** 1 Department of Molecular Microbiology, Institute of Microbiology, Biotechnology and Immunology, Faculty of Biology and Environmental Protection, University of Lodz, Banacha 12/16, 90-237 Lodz, Poland; 2 Department of Organic Chemistry, Faculty of Pharmacy with Medical Analytics Division, Medical University of Lublin, Chodźki 4a, 20-093 Lublin, Poland

**Keywords:** *N*-acylhydrazinecarbodithioates, Aminotriazole-thiones, Dermatophytes, Antidermatophytic activity, SEM, TEM, RNA-seq, Drug discovery, Medicinal chemistry, Drug development

## Abstract

Nowadays, dermatophyte infections are relatively easy to cure, especially since the introduction of orally administered antifungals such as terbinafine and itraconazole. However, these drugs may cause side effects due to liver damage or their interactions with other therapeutics. Hence, the search for new effective chemotherapeutics showing antidermatophyte activity seems to be the urge of the moment. Potassium salts of *N*-acylhydrazinecarbodithioates are used commonly as precursors for the synthesis of biologically active compounds. Keeping that in mind, the activity of a series of five potassium *N*-acylhydrazinecarbodithioates (**1a–e**) and their aminotriazole-thione derivatives (**2a–e**) was evaluated against a set of pathogenic, keratinolytic fungi, such as *Trichophyton* ssp., *Microsporum* ssp. and *Chrysosporium keratinophilum*, but also against some Gram-positive and Gram-negative bacteria. All tested compounds were found non-toxic for L-929 and HeLa cells, with the IC_30_ and IC_50_ values assessed in the MTT assay above 128 mg/L. The compound 5-amino-3-(naphtalene-1-yl)-4,5-dihydro-1H-1,2,4-triazole-5-thione (**2d**) was found active against all fungal strains tested. Scanning Electron Microscopy (SEM) revealed inhibition of mycelium development of *Trichophyton rubrum* cultivated on nail fragments and treated with **2d** 24 h after infection with fungal spores. Transmission Electron Microscopy (TEM) observation of mycelium treated with **2d** showed ultrastructural changes in the morphology of germinated spores. Finally, the RNA-seq analysis indicated that a broad spectrum of genes responded to stress induced by the **2d** compound. In conclusion, the results confirm the potential of *N*-acylhydrazinecarbodithioate derivatives for future use as promising leads for new antidermatophyte agents development.

## Introduction

Dermatophytes are microscopic fungi with a high affinity for keratin and the ability to decompose it. This group of anamorphic molds includes three genera: *Trichophyton*, *Microsporum*, and *Epidermophyton*, which are responsible for the surface mycoses of the skin and its products in humans and animals^1^. Modern living conditions have increased the likelihood of factors favoring the development of this type of infection, resulting in the elevated incidence of dermatophytoses over the years^2^. They occur in specific age groups that are particularly susceptible to infections (children, the elderly), as well as in specific social or professional groups^3,4^. The prevalence of superficial fungal infections caused by dermatophytes has been estimated at 25% among the population worldwide. After *onychomycosis*, which, by far, is the most difficult to cure, and *tinea capitis*, the most prevalent superficial mycosis in the United States and Canada is *tinea pedis* (affecting approximately 40% of the population), followed by *tinea corporis* and *tinea cruris*^5,6^. Therefore, fungal skin infections, including those caused by dermatophytes, are now referred to as civilization diseases, which are a significant public health problem^7–9^.

With such a high prevalence of superficial dermatophytoses in developed countries, there is a need for topical antifungals that are safe and effective. In turn, with the increasing use of antifungal agents for treating superficial dermatophytoses, the development of resistance remains a real possibility, such as in the case of other pathogenic fungi. Despite the availability of several groups of drugs, they act on a limited number of cellular targets, such as the plasma membrane, cell wall, nucleic acids, and cell division. Four groups of antibiotics are currently in use by dermatologists: azoles, polyenes, echinocandins, and allylamines^10^. Mechanisms of action of these antifungals, which often overlap, may contribute to the emergence of the multidrug resistance (MDR) effect of the medically significant fungi^10^. For example, according to CDC data (https://www.cdc.gov/fungal/index.html), fluconazole resistance among *Candida* strains has remained stable for about 20 years^11–13^ while increasing resistance to echinocandins, especially in *Candida glabrata*^14^, has become a significant problem. An alternative to the treatment of infections caused by resistant strains may be the use of amphotericin B, however, this drug is highly toxic to patients and is only administered as a last resort. Similarly, the problem of increasing drug resistance is observed in the case of *Aspergillus* infections, where a dozen or so of the approximately 300,000 cases of infections per year occurred with drug-resistant strains^15,16^.

In the case of dermatophytes, many reports suggest that drug resistance is recently on the rise^8^. In the treatment of dermatophytosis, a limited number of antifungal drugs are used, mainly azoles as well as allylamines, which has led to the development of drug resistance^17^. Moreover, some drugs may cause side effects such as liver damage or interact with other agents used by patients to treat certain diseases^18–21^. On the other hand, other available topical agents, such as amorolfine 5% and ciclopirox 8%, have low efficacy^22,23^. Thus, there is a need to identify new dermatophyte inhibitors which would be highly effective against treated fungal cells while having low toxicity to the patient's organism.

Potassium salts of *N*-acylhydrazinecarbodithioates are well-known precursors in the synthesis of compounds showing anticancer^24^, antimicrobial^25–27^, antitubercular^28^, antihypertensive, and diuretic activities^29^. Analyses performed previously by some members of our broad research group revealed that a series of potassium salts of *N*-acylhydrazinecarbodithioates showed antifungal potential against different *Candida* species^30^. Based on docking simulations, fungal carbonic anhydrase (CA) was proposed as one of the putative enzymatic targets for *N*-acylhydrazinecarbodithioates derivatives^31^. CAs are found in Eukarya, Bacteria, and Archea and are essential for carbon dioxide (CO_2_) sensing and metabolism^32^. As zinc-containing metalloenzymes, they are responsible for the hydration of CO_2_ to bicarbonate (HCO_3_^−^) and proton (H^+^)^33^. CAs may play an important role in the survival and colonization of human hosts by pathogenic fungi^32^. The study of Petruccelli et al.^34^ also confirmed that carbonic anhydrase (TERG_07222) belongs to the group of proteins that are important for the pathogenesis of *Trichophyton rubrum.* Hence, it is very likely that *N*-acylhydrazinecarbodithioates derivatives may also show good activity against these medically important fungi. For that reason, in this study, we investigated in vitro the antifungal potential of the previously described potassium salts of *N*-acylhydrazinecarbodithioates^31^ and their cyclic derivatives with aminotriazole-thione core against dermatophytes and other keratinolytic fungi as well as their cytotoxic and antibacterial activities. Additionally, we examined the in vitro effect of the most active compound 5-amino-4-(naphthalene-1-yl)-2,4-dihydro-3H-1,2,4-triazole-3-thione (**2d**) on the morphology of *T. rubrum* using three different microscopic tools such as white-light microscopy (WML), scanning electron microscopy (SEM) and transmission electron microscopy (TEM) and analyzed global transcriptomic changes using RNA-seq.

## Results

### In vitro antifungal activity

Five potassium *N*-acylhydrazinecarbodithioates (**1a**, **1b**, **1c**, **1d**, **1e**) and five of their *s*-triazole derivatives (**2a**, **2b**, **2c**, **2d**, **2e**) were evaluated for their activity against 14, both reference and clinical keratinolytic fungal strains. Compound **2b** was insoluble in RPMI 1640 medium in the range of the tested concentrations and DMSO restrictions, and as such, it was excluded from further studies. All compounds showed good activity against specific fungi strains. The *s*-triazole derivatives were more active than the potassium *N*-acylhydrazinecarbodithioates (**1a**, **1c**, **1d** compared to **2a**, **2c**, **2d**). The most active were *s*-triazole derivatives **2a**, **2c**, and **2d** showing the lowest MIC values against most dermatophyte strains (Table 1). At the same time, **2d** showed the broadest spectrum acting on all tested strains with MIC values in the range of 32–128 mg/L. Micromolar concentrations are listed in Table 1. Compound **2d** inhibited even the growth of *Ch. keratinophilum* CBS, which was resistant to all other compounds. The most sensitive to the tested compounds were *T. rubrum* strains with MICs in the range of 16–128 mg/L, depending on the strain. The most resistant were *Ch. keratinophilum, T. granulosum,* and *T. interdigitale CBS* however, for the latter two, MIC values of 64–128 mg/L were obtained for the best-acting compounds. Most of the compounds except **1a** showed good activity against *M. canis* strains. Surprisingly, derivatives **1e** and **2e** inhibited the growth of *M. canis* strains, especially *M. canis* 150, although they did not show activity against most *Trichophyton* species.

**Table 1:** The MIC range and geometric mean (GM) of MIC values (mg/mL) and (µM) of tested compounds **1a**–**2e** and antifungal agents amphotericin B and ketoconazole.

*Keratynolytic fungi*						MIC mg/L (μM)					
	**1a**	**2a**	**1b**	**1c**	**2c**	**1d**	**2d**	**1e**	**2e**	A*	K*
*Trichophyton rubrum CBS*	128 (535)	16 (88)	128 (425)	64 (235)	32 (149)	64 (213)	32 (132)	> 128 (> 499)	> 128 (> 646)	4 (4)	2 (4)
*Trichophyton rubrum 127/07*	> 128 (> 535)	32 (177)	> 128 (> 425)	128 (470)	32 (149)	64 (213)	32 (132)	> 128 (> 499)	> 128 (> 646)	4 (4)	2 (4)
*Trichophyton rubrum 144/10*	128 (535)	64 (353)	> 128 (> 425)	64 (235)	64 (299)	64 (213)	64 (265)	> 128 (> 499)	> 128 (> 646)	16 (17)	1 (2)
*Trichophyton rubrum 451/04*	128 (535)	16 (88)	> 128 (> 425)	> 128 (> 470)	128 (597)	> 128 (> 426)	128 (528)	> 128 (> 499)	> 128 (> 646)	4 (4)	0.5 (1)
*Trichophyton interdigitale CBS*	> 128 (> 535)	64 (353)	> 128 (> 425)	128 (470)	64 (299)	128 (426)	128 (528)	128 (499)	128 (646)	4 (4)	1 (2)
*Trichophyton interdigitale 445/10*	128 (535)	16 (88)	32 (106)	64 (235)	64 (299)	64 (213)	64 (265)	> 128 (> 499)	64 (323)	4 (4)	2 (4)
*Trichophyton granulosum 49/10*	> 128 (> 535)	128 (706)	> 128 (> 425)	128 (470)	64 (299)	128 (426)	128 (528)	128 (499)	128 (646)	4 (4)	1 (2)
*Trichophyton granulosum 175/07*	> 128 (> 535)	> 128 (> 706)	> 128 (> 425)	> 128 (> 470)	128 (597)	128 (426)	128 (528)	> 128 (> 499)	> 128 (> 646)	8 (9)	1 (2)
*Trichophyton tonsurans CBS*	> 128 (> 535)	64 (353)	> 128 (> 425)	64 (235)	64 (299)	> 128 (> 426)	64 (265)	> 128 (> 499)	> 128 (> 646)	4 (4)	0.5 (1)
*Trichophyton tonsurans 170/08*	> 128 (> 535)	32 (177)	> 128 (> 425)	128 (470)	64 (299)	> 128 (> 426)	128 (528)	> 128 (> 499)	> 128 (> 646)	4 (4)	1 (2)
*Chrysosporium keratinophilum CBS*	> 128 (> 535)	> 128 (> 706)	> 128 (> 425)	> 128 (> 470)	> 128 (> 597)	> 128 (> 426)	128 (528)	> 128 (> 499)	> 128 (> 646)	4 (4)	1 (2)
*Microsporum canis CBS*	> 128 (> 535)	128 (706)	64 (213)	64 (235)	64 (299)	128 (426)	32 (132)	128 (499)	128 (> 646)	8 (9)	1 (2)
*Microsporum canis 31*	> 128 (> 535)	128 (706)	32 (106)	128 (470)	128 (597)	128 (426)	128 (528)	128 (499)	64 (323)	4 (4)	2 (4)
*Microsporum canis 150*	128 (535)	128 (706)	64 (213)	64 (235)	128 (597)	> 128 (> 426)	128 (528)	64 (250)	16 (250)	4 (4)	4 (8)
MIC range	128—> 128 (535- > 535)	16- > 128 (88- > 706)	32- > 128 (106- > 425)	64- > 128 (235- > 470)	32- > 128 (149- > 597)	64- > 128 (213- > 426)	32–128 (132–528)	64- > 128 (250- > 499)	16- > 128 (164- > 646)	4–16 (4–17)	0.5–4 (1–8)
*N-active*	*5/14*	*12/14*	*5/14*	*11/14*	*13/14*	*9/14*	*14/14*	*5/14*	*6/14*	*14/14*	*14/14*
GM-MIC	128.0 (535.0)	50.8 (280.1)	55.7 (185.0)	87.7 (332.0)	71.2 (332.3)	94.1 (331.1)	82.0 (321.8)	111.4 (434.6)	71.8 (407.2	4.9 (5.6)	1.2 (2.4)

A*—Amphotericin B, K*—Ketoconazole.

The *N*-active indicates the number of sensitive strains among 14 analyzed dermatophyte strains.

Since antifungal agents are typically used as broad-spectrum therapeutics rather than being specific to particular fungal species, an overall geometric mean for MIC values was determined. This approach was adopted to facilitate a more comprehensive assessment of antifungal efficacy across the range of compounds tested. As expected, the lowest average MIC values were observed within the group of s-triazole derivatives (Table 1). It is worth noting an exception in the case of compound **1b**, which belongs to the potassium *N*-acylhydrazinecarbodithioates, however, it should be acknowledged that this compound was effective against only five of the 14 tested fungal strains. Within the category of s-triazole compounds, the lowest geometric mean MIC values, in descending order, were associated with compounds **2a**, **2c**, **2e**, and **2d**. However, it should be noted that only **2d** showed activity against all analyzed strains in the tested concentration range (Table 1). Taking into account both the geometric mean MIC values and the number of strains sensitive to a given compound, it becomes evident that **2a**, **2c**, and **2d** appear to be the most promising antifungal compounds.

For the compounds for which the MIC values were determined, we conducted an additional analysis to assess their fungicidal activity. This analysis involved transferring 50 µL aliquot from a plate’s well with MIC to a fresh growth medium. After an incubation period of 48 h, it was observed that only compounds **2a**, **2c**, and **2d** exhibited inhibition of fungal growth of *T. rubrum*, as well as both strains of *T. interdigitale* and *T. tonsurans* CBS (Table S1). Consequently, it can be concluded that the remaining compounds displayed fungistatic properties rather than fungicidal effects within the tested concentration range.

### In vitro antibacterial activity

To determine the antibacterial activity of the tested compounds against a set of both Gram-negative and Gram-positive bacterial strains, a broth microdilution method was performed in the concentration range of compounds from 1 to 256 mg/L^35^. For all compounds, it was impossible to determine the MIC value, defined as the lowest concentration of the drug, which prevents the visible growth of bacteria. Therefore, MIC_70_ and MIC_50_ values were determined, i.e., the concentration of the compounds that inhibit bacterial growth by 70% and 50%, respectively (Table S2). Most tested compounds did not show antibacterial activity against any bacterial strain. Only compound **1b** showed moderate activity causing 50% inhibition of growth at concentrations of 16–128 mg/L, depending on the strain. The best-acting against dermatophytes s-triazoles **2a**, **2c**, and **2d** did not show simultaneous antibacterial activity, only compound **2d** caused 50% inhibition of E. *coli* growth at a concentration of 32 mg/L.

### Cell viability assay

The toxicity of the tested compounds, expressed as inhibition of cell viability of murine fibroblasts L929 and human tumor HeLa cell lines, was determined by the MTT assay in the compounds’ concentration of ranging from 0.5 to 128 mg/L. To estimate the safety of the tested derivatives for eukaryotic cells, IC_50_ and IC_30_ values were determined, i.e., concentrations causing 50% and 30% inhibition of cell viability, respectively. The latter parameter is considered a non-toxic concentration of the drug. All compounds showed no toxic effect (Table 2) or induction of morphological changes (data not shown) in the cells of the analyzed cell lines in the tested concentration range. Only in the case of compounds **2a** and **1b** was it possible to determine IC_30_ values at 128 and 100 mg/L, respectively. It is worth noting that the obtained MIC values against sensitive fungal strains were lower than the IC_50_ values for the mammalian cell lines, and the selectivity index (SI) for all compounds reached the value above 1, and in the case of the most fungistatic compounds close to or above 8 (Table S3).

**Table 2:** Effect of **1, 2 a–e** on the viability of L929 and HeLa cell lines expressed as a compound concentration inhibiting cell growth by 30% (IC_30_) and 50% (IC_50_) [mg/L].

	Cell viability assay			
	**L929**		**HeLa**	
	IC30	IC50	IC30	IC50
1a	> 128	> 128	> 128	> 128
2a	128	> 128	> 128	> 128
1b	100	> 128	100	> 128
1c	> 128	> 128	> 128	> 128
2c	> 128	> 128	> 128	> 128
1d	> 128	> 128	> 128	> 128
2d	> 128	> 128	> 128	> 128
1e	> 128	> 128	> 128	> 128
2e	> 128	> 128	> 128	> 128
Amphotericin B	16	64	> 128	> 128
Ketoconazole	64	100	64	128

All compounds were less toxic than the commonly used antifungals, amphotericin B and ketoconazole, on the L929 cell line and ketoconazole on the HeLa cell line in the meaning of concentration used, however, the SI was much higher for these drugs than for tested compounds because of lower MIC values obtained for analyzed fungal strains. The results confirmed that the tested potassium *N*-acylhydrazinecarbodithioates and their *s*-triazole derivatives were safe for mammalian cells in vitro in concentrations inhibiting the growth of dermatophytes and may be considered as potential leads in the development of antifungal agents. According to the above results, out of all the compounds analyzed, we chose the **2d** for further analysis because it appeared to be the most promising candidate among the compounds tested.

### Molecular docking

**Figure 1:**
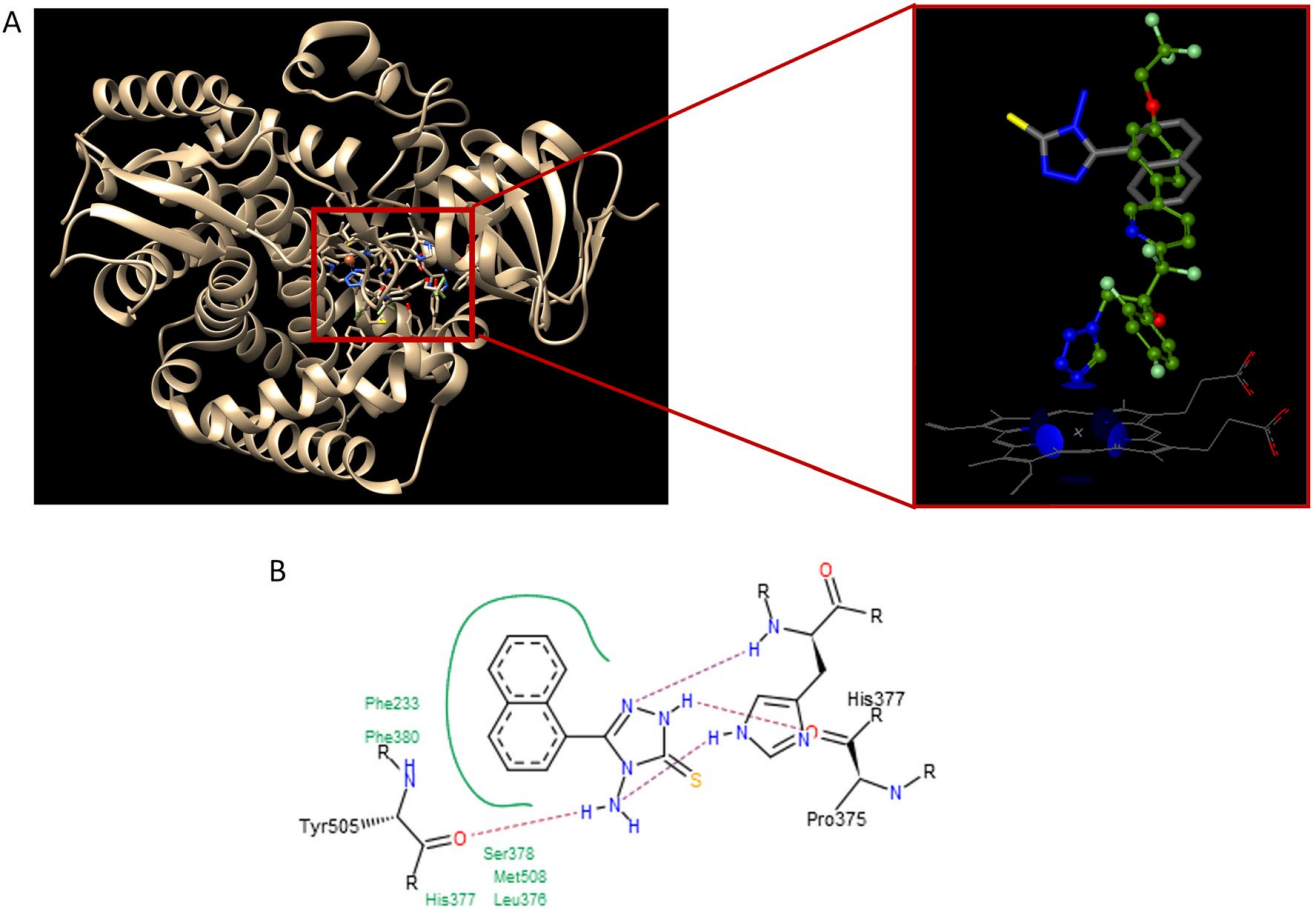
(**A**) The binding mode of **2d** into the active site of CYP51 (PDB id: 5TZ1) (left) and the docked conformation of **2d** (grey stick) superimposed with the crystal conformation of native inhibitor VT1161 (green stick) (right). Nitrogen atoms are blue, oxygen atoms are red, and sulphur atom is yellow. (**B**) PoseView image for a docked pose of **2d**. Hydrophobic interactions are displayed as green contact curves and the H-bonds are presented as purple dashed lines.

Using the crystal structure of *C. albicans* CYP51 (CACYP51) in complex with the tetrazole-based antifungal drug candidate VT1161 as a template (PDB 5TZ1)^36^, we conducted a docking study of the aminotriazole-thione **2d** to assess its possible potential for CYP51 inhibition. According to docking results, the binding score is − 26.7 for 2d and − 21.5 for VT1161, reflecting the higher binding affinity of **2d** compared to the native inhibitor VT1161. As shown in parts A and B of Fig. 1, the naphthyl group of **2d** is positioned in the S4 pocket of CACYP51^37^ at the same hydrophobic binding cleft as the phenoxy group of native inhibitor VT1161. Its binding mode is stabilized by close hydrophobic contacts with a set of 6 residues and all are identical to those interacting with VT-1161. These include Met-508 from β4 hairpin, Phe-380 from β1-4 strand, Phe-233 from helix F”, and three residues from K/β1-4 loop (Leu-376, His-377, as well as Ser-378 recognized as substantial residues in the design of CACYP51 inhibitors). For the triazole group of **2d**, in turn, three hydrogen bonds were predicted between its amino group at the N4 position and the carbonyl group of Tyr-505, the N2 nitrogen atom and the carbonyl group of Pro-375, as well as between the N1 nitrogen atom and the amino group of conserved residue (His-377) in all CYP51 enzymes from the *Candida* genera^36^, In contrast to long-tailed native inhibitor VT1161, neither coordination bond with the prosthetic heme iron through the triazole ring of **2d** nor its close contacts with the heme-binding core region were recorded. Additionally, no close interactions of **2d** with Tyr-132, recognized as a crucial residue to the antifungal ability^38^ were observed. Thus, we can conclude that although short-tailed **2d** can inhibit CYP51 activity by competing with the sterol substrate for the space within the enzyme-active site, it cannot affect the iron potential to be reduced^36^. Based on the binding mode of known CYP51 inhibitors deposited in Protein Data Bank^39^ (PDB id: 7RYX, 5ESG, 5ESH, 5ESK, 8DL4, 5EAB, 8EAC, 5EAD, 5EAE, 5EAF, 5EAG, 5EAH, 5FSA, 5FRB, 7RYA, 5JLC, 4ZDY, 4ZE1, 4ZE2, 4UYL, 6CR2, 5ESL, 5V5Z, 5UL0, 6E8Q, 5EQB), particularly along with the binding mode of short-tailed triazole-based antifungals fluconazole (PDB 5ESJ, 5ESE, 5ESF, 4WMZ, 4ZDZ, 4ZE3, 5ESM) and voriconazole (7RY9, 7RYB, 7RY8, 4ZE0, 5HS1, 4UYM), as well as the structural features of the triazoles reported in scientific literature^37,40–44^ we conclude that the mode of the inhibition observed for the short-tailed aminotriazole-thione **2d** does not fully correspond with key structural features of known CYP51 azole-based inhibitors.

### Cell viability by resazurin reduction assay (RRA) and microscopic observation of *T. rubrum*

**Figure 2:**
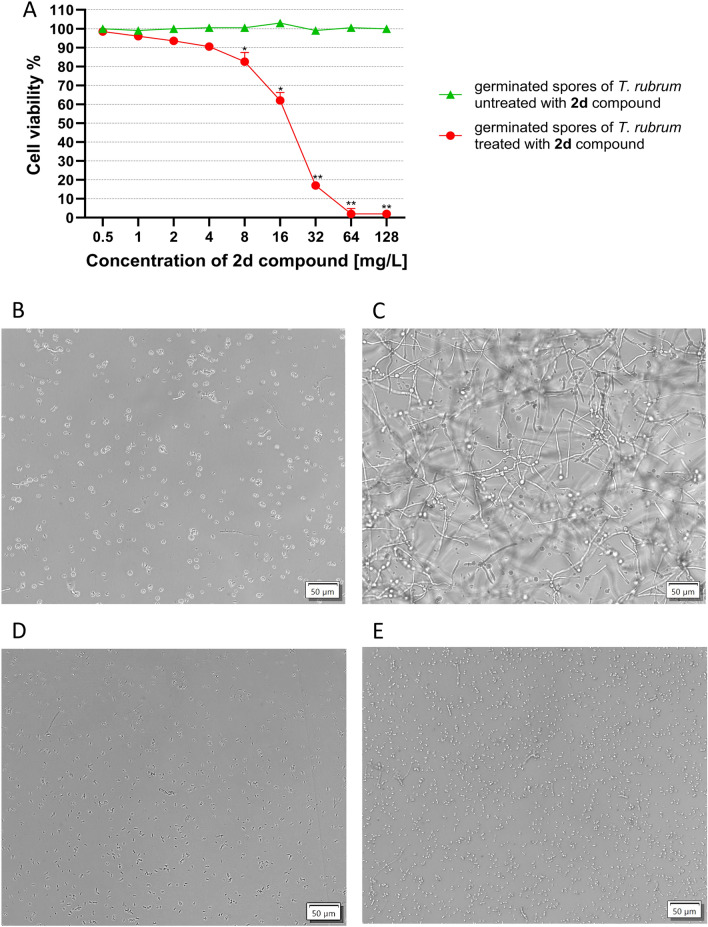
Resazurin reduction assay (RRA) and white-light microscopy observation of *T. rubrum* CBS 120358. (**A**) Viability of germinated spores of *T. rubrum* measuring using RRA after 24h incubation with different concentration **2d** compound. Data are expressed as mean ± S.E.M (n = 3), *p < 0.05 and **p < 0.01 vs. control. Bonnefroni test. (**B**) Germinated spores untreated with 2**d** compound, (**C**) Growth of hyphae from untreated with **2d** compound germinated spores after 24 h of incubation; (**D**) germinated spores treated with **2d** compound (1 × MIC). (**E**) No mycelia growth from germinated spores treated with **2d** compound (1 × MIC) at 24 h. Scanning objective × 40 and Eyepiece × 10 = Total magnification, × 400.

Germinated spores of *T. rubrum* treated with **2d** compound in the tested concentration range (128–0.5 mg/L) resulted in filamentation inhibition > 80% at a concentration of 32 mg/L (MIC) vs. control that contained germinated spores of *T. rubrum* untreated with **2d** compound. *T. rubrum* was incapable of filamentation due to the action of the 2d compound at a concentration of 32 mg/L, equal to MIC (Table 1, Fig. 2A).

White-light microscopy (WLM) observation of *T. rubrum* growing from untreated with **2d** compound germinated spores (Fig. 2B) showed typical hyphae, as shown in Fig. 2C. A 24 h incubation of germinated spores of *T. rubrum* treated with **2d** compound (Fig. 2D), added at a concentration equal to MIC, resulted in no filamentation (Fig. 2E).

**Figure 3:**
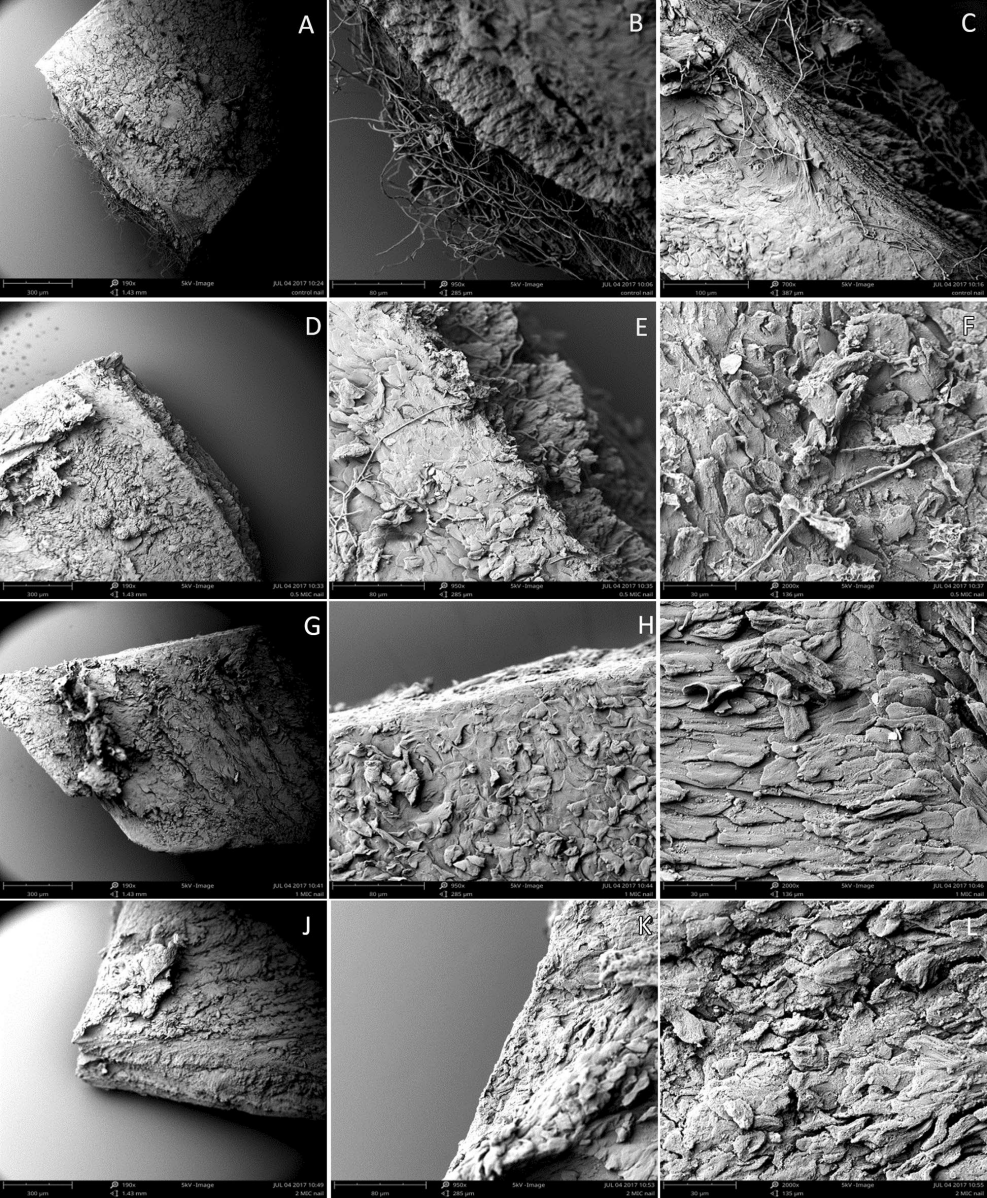
Scanning-electron microscopy showing mycelial structures of *T. rubrum* CBS 120358 cultured on nail fragments for 24 h at 28 °C: (**A–C**) Control without drug, (**D–F**) treatment with **2d** compound at 0.5 × MIC, (**G–I**) treatment with **2d** compound at 1 × MIC, (**J–L**) treatment with **2d** compound at 2 × MIC. Bars: 300 μm (**A, C, E, G**); 100 μm I; 80 μm (**B, E, H, K**); 30 μm (**F, I, L**).

To investigate the growth of *T. rubrum* on nail samples in the presence of compound **2d**, scanning electron microscopy (SEM) was used to observe mycelia growing within the nail plate. Figure 3A–C (untreated control) present the typical structure of hyphae growing on the nail fragment. SEM showed that the infected nail samples were significantly damaged. Dissociated layers and a thin layer of keratinocytes were observed. In contrast, anti-dermatophyte effects of the **2d** compound at concentrations equal to 0.5 MIC, 1 × MIC, and 2 × MIC after 24 h incubation are shown in Fig. 3D–F, G–I, and J–L, respectively. It can be observed that while 0.5 × MIC only moderately limited the growth of the fungus, both 1 × MIC, and 2 × MIC concentrations of the tested compound resulted in inhibited development of *T. rubrum* mycelium.

**Figure 4:**
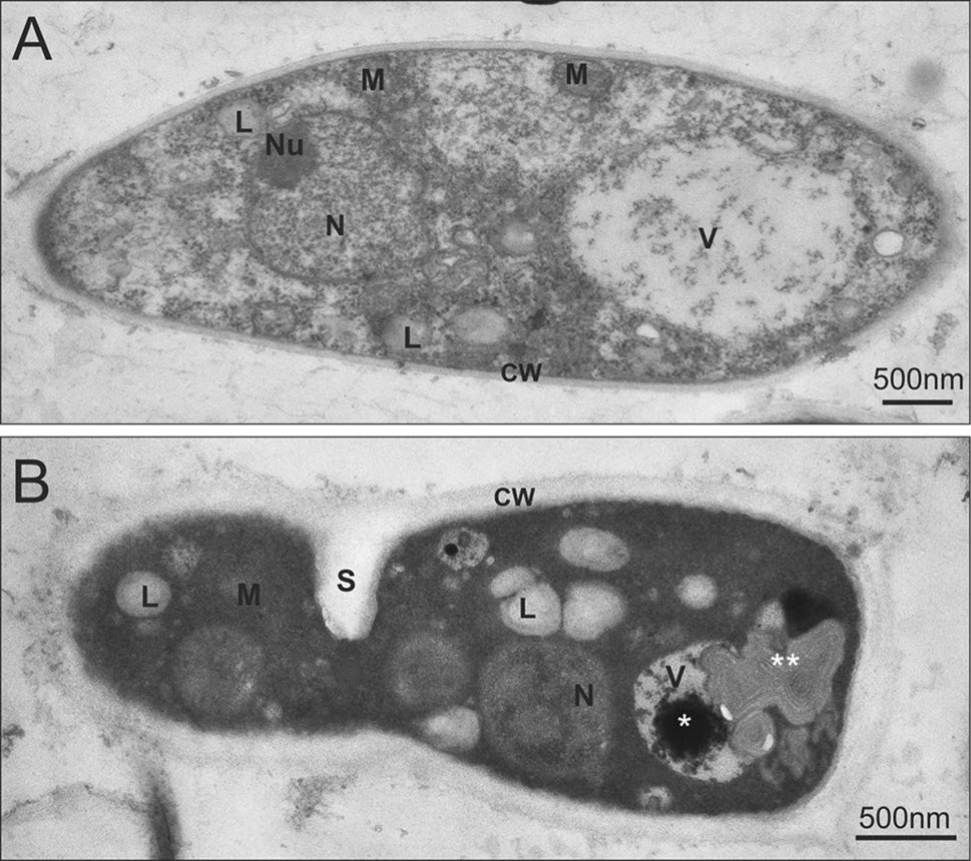
TEM micrographs of control (**A**) and drug-treated germinated spores (**B**) of *T. rubrum* CBS 120358 24 h after **2d** compound (32 mg/L) application. Bar = 500 nm; **CW**—cell wall; **L**—lipid droplet; **M**—mitochondrion; **N**—nucleus; **Nu**—nucleoli; **S**—septum; **V**—vacuoles, *—electron-dense material in vacuole; **—myelin-like structure.

Transmission electron microscopy (TEM) observation of untreated *T. rubrum* germinated spores revealed typical cytoplasmic components, such as thin cell wall, large vacuoles with flocculent content, nuclei with nucleoli, oval mitochondria with few cristae (Fig. 4A). Meanwhile, the germinated spores treated with 1 × MIC of **2d** compound after 24 h showed condensed organelles such as nucleus and mitochondria, with the electron-dense material and myelin-like structure in the vacuoles, as well as numerous, large lipid droplets within electron-dense cytoplasm (Fig. 4B).

### RNA-seq analysis of *T. rubrum* transcriptome in response to the 2d compound

The gene expression profile of *T. rubrum* exposed to the **2d** compound was assessed using next-generation sequencing. A total of over 200 million sequence reads were obtained, which corresponded to 4 libraries and consisted of 50–150 base pairs. Alignment was performed against the reference genome of *T. rubrum* available at the Broad Institute using the Bowtie tool^45^. DEGs with significant fold change at least ≥ 1.5 and at a probability level of p < 0.05 were applied to define the expression levels of genes. In total, 3461 genes were modulated in response to the **2d** compound compared to the control at 24 h incubation time (Table S4). A global heatmap representation of gene expression profiles in *T. rubrum* exposed to the **2d** compound compared to the control condition revealed considerable modulation of gene expression (Fig. S1). A volcano plot was created to assess the distribution of log_2_FC values relative to the corrected *p*-value (Fig. S2). In turn, an MA plot was used to represent the DEGs of the **2d** compound treatment relative to the control (Fig. S3).

### Functional categorization of *T. rubrum* genes involved in the response to 2d compound

**Figure 5:**
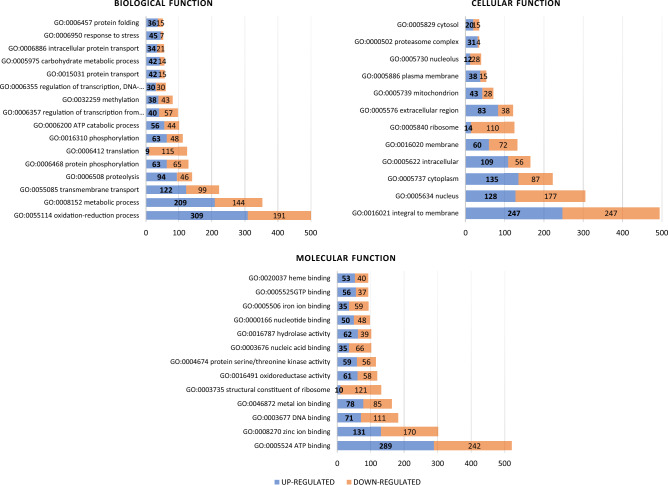
Functional annotation of the most representative genes modulated by *T. rubrum* CBS 120358 in response to the **2d** compound based on Gene Ontology (GO). Blue and orange bars indicate up-and-down-regulated genes, respectively.

Differentially expressed genes were analyzed according to the classification of gene function within the gene ontology categories (GO) (Fig. 5). The majority of annotations for DEGs were assigned into three main functional categories: cellular components, molecular function, and biological process. Among the biological process categories, the oxidation–reduction processes (GO:0055114), metabolic processes (GO:0008152), and transmembrane transport (GO:0055085) were the most highly represented. Integral components of the membrane (GO:0016021), nucleus (GO:005634), and cytoplasm (GO:0005737) represented the major proportion of the cellular component categories, while ATP binding activity (GO:0005524) and zinc ion binding activity (GO:0008270) were the most abundantly represented among the various molecular functions (Fig. 5).

### Validation of RNA-seq by qRT-PCR

To validate Illumina RNA-seq gene expression data, a total of 12 genes (Table S5) were selected for expression profile analysis by qRT-PCR. Changes in expression levels determined by qRT-PCR were compared with the results of the RNA-seq expression analysis. As expected, the qRT-PCR results were similar to the RNA-seq results, with identical up- or downregulation trends for each gene (Fig. S4), suggesting the reliability and accuracy of the RNA-seq expression analysis.

### Expression profiles of genes related to ergosterol biosynthesis

**Figure 6:**
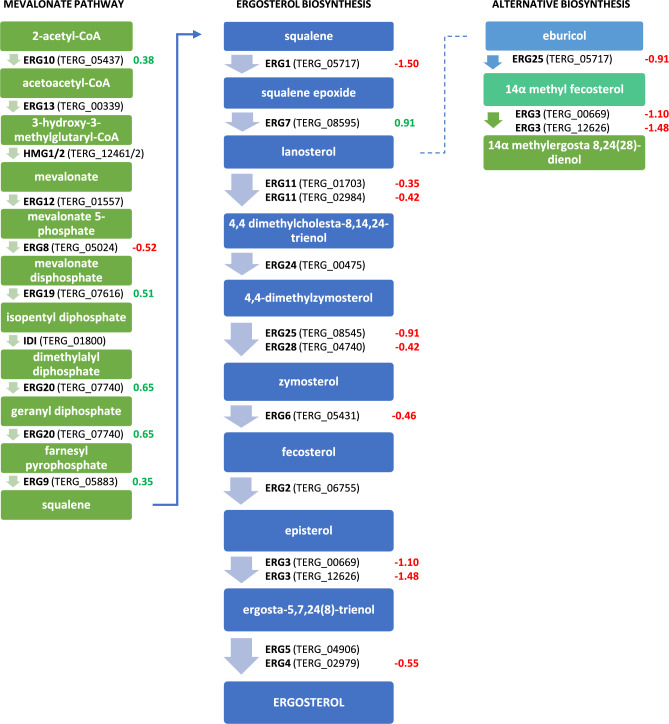
Mevalonate and ergosterol biosynthesis pathways modulated after exposure of *T. rubrum* CBS 120358 to the **2d** compound (log_2_FoldChange). Green values correspond to up-regulated genes and red values correspond to down-regulated genes. Adapted from the article Martins MP et al. Comprehensive analysis of the dermatophyte *Trichophyton rubrum* transcriptional profile reveals dynamic metabolic modulation. *Biochem J* (2020) 477 (5): 873–885.

We analyzed the essential genes involved in the ergosterol biosynthesis pathway. The results, revealed in Fig. 6, show that 19 genes (6 up-regulated and 13 down-regulated) were differentially expressed during the exposure of *T. rubrum* to the **2d** compound. The expression of ERG11, ERG25, ERG28, ERG6, ERG4, and ERG4 genes involved in the late pathway were repressed under the conditions evaluated.

### Expression profiles of genes related to the cell wall and involved in transmembrane transport

The ABC (ATP-binding cassette transporter) and MFS (Major Facilitator Superfamily transporter) transporters play an important role in fungal survival while the cell wall protects them against environmental stress. We have investigated 37 DEGs involved in transmembrane transport and 45 DEGs related to cell wall integrity in response to exposure to the **2d** compound (Table S6). Among the membrane transporters, 23 genes were upregulated, and 14 genes were downregulated. In turn, in the case of cell wall-related genes, 26 were upregulated, while 19 genes were downregulated (Table S6).

## Discussion

Dermatophyte infections pose a serious dermatological and epidemiological problem. Despite the many antifungal agents clinically available, including amphotericin B, fluconazole, voriconazole, itraconazole, and posaconazole, as well as the high level of drug susceptibility within dermatophyte species, this favorable situation may change rapidly^46–50^. Thus, there is an urgent need to search for new compounds with antifungal activity that could become handy tools in eradicating dermatophyte infections. Research of this type is being conducted in two directions. The first is the search for new classes of compounds that would exhibit the desired high antifungal activity, often aiming for completely new molecular targets. This type of approach offers a relatively good chance of avoiding already developed drug resistance mechanisms. However, it requires a great deal of effort at both the design, synthesis, and testing stages of the compounds developed in this way. The second approach consists of modifying the already proven backbones of antimicrobial compounds by adding suitable substituents, thereby providing these agents with new, additional activities or making them "invisible" to drug resistance mechanisms. The present study examined antifungal, antibacterial, and cytotoxic activities of five potassium N-acylhydrazinecarbodithioates (**1a**, **1b**, **1c**, **1d**, **1e**) and five of their *s*-triazole derivatives (**2a**, **2b**, **2c**, **2d**, **2e**) against different keratinolytic fungi. Both series of compounds revealed a lack of antibacterial activity (Table S2) however, they were potent agents against reference and clinical strains of keratinolytic fungi. Among the species tested, *Chrysosporium keratinophilum* was the most resistant to the compounds tested. Only one compound, 5-amino-4-(naphthalene-1-yl)-2,4-dihydro-3H-1,2,4-triazol-3-thione (**2d**), inhibited the growth of all tested fungi within MIC range of 32–128 mg/L. Moreover, this compound, which is the cyclic *s*-triazole form of *N*-acylhydrazinecarbodithioates, was not toxic to L929 and HeLa cell lines (Table 2).

Using microscopic analyses such as WLM, SEM, and TEM, we demonstrated the morphological changes of *Trichophyton rubrum* caused by 5-amino-4-(naphthalene-1-yl)-2,4-dihydro-3H-1,2,4-triazol-3-thione (**2d**). The inhibitory effect of **2d** can be observed in Figs. 2, 3, and 4. Resazurin reduction assay (RRA) as well as WML demonstrated inhibition of *T. rubrum* filamentation from germinated spores previously treated with **2d** compound at 32 mg/L (Fig. 2). SEM is a very suitable tool for observation of the spatial interaction between dermatophytes and the nail plate^46^. Our SEM results showed that the nail plate, infected by *T. rubrum,* was damaged to a layered form with a very irregular shape. Hyphae of *T. rubrum,* which were straight and smooth, without wrinkles on their surface, penetrated the thin layers of keratinocytes (Fig. 3B, C). Treatment with **2d** at a concentration equal to 0.5 × MIC during 24 h resulted in moderate growth inhibition and morphological changes, such as distortion of the hyphal elements (Fig. 3E, F), while *T. rubrum* exposed to **2d** at 1 × MIC and 2 × MIC showed a lack of mycelia growth on the nail plate.

The TEM observation of the untreated *T. rubrum* germinated spores has shown the correct morphology of the cell wall, cell membrane, and organelles such as nuclei, mitochondria, or vacuoles (Fig. 4). In contrast, after 24 h exposition to the **2d** at 1 × MIC (32 mg/L), disruption of intracellular material was observed. The activity of antidermatophyte agents such as amphotericin B, ketoconazole, itraconazole, or terbinafine, is based on four mechanisms of action: (1) disturbance of the cell wall synthesis, (2) inhibition of nucleic acid synthesis (3) inhibition of proteins synthesis, (4) disturbance of cell membrane components synthesis. According to the literature^46–50^, the influence of antifungal compounds mentioned above can lead to morphological changes in the cell wall structure or the appearance of electron-dense granules in the cell wall, which is the result of the action of ergosterol synthesis inhibition. Vanden Bossche et al.^51,52^ described the granular structures in the cell wall as agglomerates of intermediates in sterol metabolism that accumulate during the process of ergosterol synthesis inhibition. Our TEM observation of *T. rubrum* germinated spores after treatment with the **2d** compound did not reveal morphological changes, such as the presence of granular electro-dense structures between the plasma membrane and the cell wall. However, some cell wall changes and degeneration of organelles (Fig. 4B) were observed, which may indirectly indicate that the mechanisms of action of 5-amino-4-(naphthalene-1-yl)-2,4-dihydro-3H-1,2,4-triazol-3-thione (**2d**) is related to the disturbance of ergosterol synthesis.

To understand the molecular mechanisms by which *T. rubrum* responds to exposures with the **2d** compound, we performed a wide-transcriptome analysis using the RNA-seq method. Although some genes related to fungal growth during exposure to the antifungal compounds^53,54^, can be modulated after 3, 6, or 12 h, we decided to check global expression after 24 h, when the mycelium should be adapted to external stress. We observed a few categories of genes modulated by 5-amino-4-(naphthalene-1-yl)-2,4-dihydro-3H-1,2,4-triazol-3-thione such as those, products of which are involved in lipid metabolism (ergosterol biosynthesis pathway), transmembrane transport, building integral components of the cell wall as well as an oxidative stress response, which is typically a cellular adaptive response to stress.

Antifungal agents such as azoles are responsible for inhibiting the activity of the enzyme lanosterol 14-alpha-demethylase (*erg*11), a cytochrome P450–dependent enzyme that converts lanosterol to ergosterol. Inhibition of this enzyme results in the destabilization of fungal cell membranes. The exposure of *T. rubrum* to ketoconazole or itraconazole resulted in the upregulation of the essential genes of the ergosterol biosynthesis pathway (ERG): *erg*3, *erg*4, *erg* 6, *erg*11, and *erg*25^55,56^. In turn, ERG gene up-regulation is responsible for azole resistance in *T. rubrum*^55,56^. However, in our study, eight genes involved in the biosynthesis of ergosterol, i.e. *erg*1, *erg*3, *erg*4, *erg*6, *erg*8, *erg*11, *erg*25, and *erg*28, were downregulated (Fig. 6). Similar results were described by Mendes et al.^54^ who observed the repression of *erg*3 and *erg*4 genes in response to exposure of *T. rubrum* to undecanoic acid (UDA). *Erg*4 and erg6 downregulation in *T. rubrum* was also observed in response to acriflavine exposition^53^. Considering previously described data, the results obtained by our group may suggest that 5-amino-4-(naphthalene-1-yl)-2,4-dihydro-3H-1,2,4-triazol-3-thione (**2d**), similarly to the undecanoic acid^54^ and acriflavine^53^, is an antifungal agent which interferes with the biosynthesis of membrane components and may lead to the reduction of the level of ergosterol and in turn lead to the limitation of *T. rubrum* growth during drug exposure (Fig. 2). Moreover, **2d** compound, like undecanoic acid^54^ amphotericin B^55,57^ and itraconazole^58^ may be defined as “oxidative stress drugs” because of their ability to upregulate some genes in *T. rubrum* which products are related to the oxidative stress response, such as cytochrome c peroxidase (TERG_01463; log_2_fold change: 0.4439), glutathione S transferase (TERG_03390, log_2_fold change: 1.3317; TERG_02041, log_2_fold change: 0.9272; TERG_04960, log_2_fold change:1.4150), peroxidase (TERG_01463, log_2_fold change: 0.4439), superoxide dismutase (TERG_08969, log_2_fold change: 0.1578; TERG_07262, log_2_fold change: 1.1030; TERG_04335, log_2_fold change: 0.6904), mitogen-activated protein kinase (TERG_00595, log_2_fold change: 0.4664) (Table S5). Based on the work of Mendes et al.^54^ we can speculate that the cellular antioxidative system may be a target for the antifungal action of 5-amino-4-(naphthalene-1-yl)-2,4-dihydro-3H-1,2,4-triazol-3-thione similar to other antifungal compounds such as the undecanoic acid^54^ amphotericin B^54,57^ and itraconazole^58^. Superoxide dismutases play an important role in protecting the cell from the damaging action of reactive oxygen species (ROS), while glutathione S-transferases (GTSs) are responsible for detoxifying xenobiotic agents^54^. The correlation between the repression of genes related to the ergosterol biosynthesis pathway, which is important for the protective function during oxidative stress and overexpression of genes related to oxidative stress may suggest that the **2d** can sensitize *T. rubrum* cells to oxidative metabolites.

The exposure of *T. rubrum* to the **2d** also elicited changes in the expression of genes related to transport (Table S6), such as ABC and MFS transporters, which are mainly involved in drug resistance^53^. Twenty-four genes, identified using RNA-seq analysis, were upregulated, suggesting that their products may have an affinity to the **2d** and may be involved in the efflux of the compound from the cells of *T. rubrum*. On the other hand, the *TruMdr*2 gene encoding multidrug Mdr2 transporter of the ABC family responsible for resistance to antifungal drugs, as well as the other 12 genes, were downregulated in the presence of **2d** (Table S6). Perrsinoti et al.^53^ observed the downregulation of the *TruMdr*2 gene in *T. rubrum* after exposure to sublethal doses of acriflavine, explaining that this antifungal agent interferes with processes involved in the cell detoxification and general growth of *T. rubrum*^53^ what makes this gene an important target for novel antifungal agents.

The cell wall of dermatophytes plays a significant role in adhesion to the host tissue and is responsible for the cell’s shape and rigidity. On the other hand, the drug-induced changes in the cell wall organization or functional disruption of its components may reduce its functionality, leading even to the death of the fungus. For that reason, the cell wall is a promising target for drug discovery. RNA-seq analysis of *T. rubrum* presented in this work revealed modulation of forty-five cell wall-related genes (Table S6) after 24 h exposure to the 5-amino-4-(naphthalene-1-yl)-2,4-dihydro-3H-1,2,4-triazol-3-thione. Particular attention should be paid to several genes belonging to this group, such as those encoding hydrophobin and acetyltransferases of the GNAT (Gcn5-related *N*-acetyltransferase) family, as well as GPI (Glycosylphosphatidylinositol)-anchored cell wall proteins.

Hydrophobin belongs to the group of surface-active proteins produced by filamentous fungi^59^. They are responsible for fungal growth and their interaction with the environment. The presence of hydrophobin on the spore surface makes the pathogen-associated molecular patterns (PAMP), i.e., sets of pathogen-related molecules containing characteristic immunogenic microbial structures, unrecognizable by cells of the innate immune system^59^. Our results revealed that the hydrophobin gene (TERG_04234) is downregulated in response to **2d**, which may lead to a decrease in cell wall hydrophobicity and perhaps to the change of the cell wall structure (Fig. 4)^60,61^. Inhibition of the hydrophobin gene of *T. rubrum* in the presence of the **2d** compound may also lead to an increase in the level of the host’s immune response. The **2d** compound also represses a few genes that encode enzymes belonging to the GNAT family of acetyltransferases that are involved in posttranslational modification (Table S6)^61^, as has been observed for acriflavine^53^ and undecanoic acid^54^. It has been reported that these proteins have been identified in *Aspergillus flavus*^62,63^, *Candida albicans*^64^, *Saccharomyces cerevisiae*^65^ *Cryptococcus neoformans*^66^, *Fusarium graminearum*^67^ and are involved in the growth, development, regulation of secondary metabolism, regulation of transcriptional responses to various environmental stimuli, such as oxidative stress, heat, cold, and low nutrients availability^61^. The obtained results may indicate that a few members of the GNAT family have the potential as antifungal drug targets. *T. rubrum* exposition to **2d** led to the inhibition of GPI-anchored protein (Table S6). Analyses performed in *S. cerevisiae*^68^, *Candida albicans*^69^, and *Aspergillus fumigatus*^70^ revealed that these proteins play a key role in cell wall morphogenesis but also are involved in cell adhesion, cell-wall metabolism, and host immune response^70^. Thus, we speculate that the downregulation of the GPI-anchored protein genes in response to the **2d** compound can lead to the suppression of *T. rubrum* hyphal growth or its adherence to the host tissue.

Potassium salts of *N*-acylhydrazinecarbodithioates used in this study (**1a–e**) were identified and synthesized based on their inhibitory activity against fungal carbonic anhydrase (CA) reported in the docking studies by Siwek et al.^31^. They used the X-ray crystallographic structure of the *Cryptococcus neoformans* CA, which served as a model of the active site of the *Candida* enzyme. The docking results revealed that all tested salts bound to Zn (II) ion within the CA active site and were stabilized by hydrogen bonds of a sulfur anion with Gly128, but also by at least one additional hydrogen bond between the compound Asp70, Cys68, or Ser71, and close van der Waals contacts with Arg72, Gly128, Asp70, Cys127, Ala69, Val92, Leu149, Ser71, Cys68, or Ile131. The results of docking studies, together with the revealed moderate antifungal activity of N-acylhydrazinecarbodithioates potassium salts against three strains of *Candida albicans* and *Candida parapsilosis,* led to the hypothesis about the mechanism of action of these compounds as CA inhibitors. CA is an enzyme involved in the CO_2_/HCO_3_^–^ balance in multiple biological pathways and may play an important role in the growth, pathogenicity, and virulence of bacteria and pathogenic fungi, including^34^. The results presented in this paper confirmed the antifungal ability of tested *N*-acylhydrazinecarbodithioates potassium salts **1a–e** to inhibit also the growth of keratinolytic fungi from the *Trichophyton* and *Microsporum* genus. However, the cyclic derivatives of *N*-acylhydrazinecarbodithioates potassium salts, s-triazoles **2a–e**, showed significantly better antifungal activity, suggesting different or additional mechanisms regarding CA inhibition. The transcriptomic analysis of the best-acting **2d** revealed a slight upregulation (log_2_fold change: 0.29) of the CA gene (TERG_07222). That can be explained by the fact that due to the inhibition of the CA activity by compound binding at the enzyme active site, the cell increases its gene expression and CA synthesis to maintain the enzyme function at the proper level. However, comparing the differences in expression of the CA gene to other genes described above, it seems that it does not play a pivotal role in the mechanism of action of the tested compound **2d**. These findings received support from our docking studies (Fig. 1) performed using the HYDE scoring function^71–73^ with beta-carbonic anhydrase of the other filamentous fungus *Aspergillus fumigatus* (PDB id: 6JQE^i^), which also suggested a mechanism of action other than CA inhibition.

## Conclusions

Developing novel antifungal agents with high efficiency against dermatophytes is currently an urgent need. We investigated novel derivatives of N-acylhydrazinecarbodithioates against the most common keratinolytic fungi, especially dermatophytes. Our results show the potent in vitro activity of 5-amino-4-(naphtalene-1-yl)-2,4-dihydro-3H-1,2,4-triazol-3-thione (**2d**) against *T. rubrum.* The microscopic observations, as well as large-scale sequencing of the *T. rubrum* transcriptome, revealed that the stress condition resulting from the response to exposure to compound **2d** affected mainly the expression of the genes involved in the cell membrane and the cell wall remodeling but also oxidative stress response and pathogenesis, similarly to the undecanoic^54^ and acriflavine^53^. The original assumption that the **2d** compound is an inhibitor of carbonic anhydrase (CA), which is involved in the growth process, was not confirmed by the obtained results. Moreover, molecular docking also suggests that the mechanism of action of the **2d** compound is other than CA inhibition.

## Materials and methods

All methods were performed following the relevant guidelines and regulations.

### Fungal strains used in this study

The list of keratinolytic fungi used in antifungal susceptibility testing is presented in Table 3. Strains were cultivated on Sabouraud agar slants for 14 days at 28 °C, maintained on the slopes of Sabouraud-dextrose agar, and sub-cultured every 14 days. Colonies were covered with 5 mL of sterile water supplemented with 0.1% Tween-20. The spores were carefully rubbed with a sterile wooden stick, filtered using Falcon 40 µm Cell Strainer (Corning, New York, USA) to remove hyphae, and then transferred into 20 mL of YG medium containing 0.5% yeast extract as well as 2% glucose and cultivated for 3 h at 34 °C with agitation. Next, according to the European Committee on Antimicrobial Susceptibility Testing (EUCAST; E. Def 9. 3. 2)^35^ guidelines, inoculum was prepared with a turbidity of 0.5 McFarland units^74^, which equals a concentration of 2–5 × 10^6^ CFU/mL (number of spores−colony forming units per milliliter)^35^, and then diluted 1:10 to obtain the final working concentration of germinated spores 2–5 × 10^5^ CFU/mL used for antifungal activity assay, WML, SEM and TEM microscopic observation, as well as RNA-seq analysis.

**Table 3:** Strains of keratinolytic fungi used in antifungal susceptibility testing.

	Keratynolytic fungi	Collection
Reference strains	*Trichophyton rubrum* CBS 118892	Westerdijk Fungal Biodiversity Institute (formerly CBS-KNAW Collections), the Netherlands
	*Microsporum canis* CBS 113,480	
	*Trichophyton tonsurans* CBS 112818	
	*Trichophyton interdigitale* CBS 120357	
	*Chrysosporium keratinophilum* CBS 104.62	
Clinical strains	*Trichophyton rubrum* 144/10	Mycological and Venereological Laboratory, Biegański Hospital, Łódź
	*Trichophyton rubrum* 451/04	
	*Trichophyton rubrum* 127/07	
	*Trichophyton granulosum* 49/10	
	*Microsporum canis 31*	Sub-Department of Veterinary Microbiology, Institute of Biological Bases of Animal Diseases Faculty of Veterinary Medicine University of Life Sciences in Lublin
	*Microsporum canis 150*	DERMED Medical Center, Łódź
	*Trichophyton granulosum* 175/07	
	*Trichophyton tonsurans* 170/08	
	*Trichophyton interdigitale*	

### Synthesis

Two series of compounds (Table 4), potassium salts of *N*-acylhydrazinecarbodithioates (1) and those of their aminotriazole-thione derivatives (2), were synthesized according to the known procedure^31,75^, schematically presented in Table S7. Potassium salts of *N*-acylhydrazinecarbodithioates (1) were prepared through a one-step procedure via the treatment of an ethanolic potassium hydroxide solution of the corresponding carboxylic acid hydrazide (R_1_CONHNH_2_) with carbon disulfide. The reaction of potassium salts of *N*-acylhydrazinecarbodithioates (1) with hydrazine yielded the aminotriazole-thiones (2). All reagents and solvents of analytical grade or higher were purchased from commercial sources and were used without purification unless otherwise stated. NMR spectra were recorded using Bruker Avance spectrometer (300 MHz). Analytical thin-layer chromatography was performed on Merck60F254 silica gel plates (Darmstadt, Germany) and visualized by UV irradiation (254 nm). Melting points were determined on a Fischer–John's block, the reported values are uncorrected.

**Table 4:** Synthetic route for N-acylhydrazinecarbodithioates (1) and their s-triazole’s derivatives (2), and the list of compounds used in the present study.


R	Potassium *N*-acylhydrazinecarbodithioates	s-triazoles
Pyrrol-2-yl	1a	2a
Isoquinolin-3-yl	1b	2b
4-methyl-thiadiazol-5-yl	1c	2c
Naphthyl-1-yl	1d	2d
Thiophen-2-yl	1e	2e

### General procedure for the synthesis of potassium *N*-acylhydrazinecarbodithioates (1)

Corresponding carboxylic acid hydrazide (0.01 mol), solubilized in absolute ethanol (25 mL), was treated with carbon disulfide (1 mL) in the presence of solid potassium hydroxide (0.015 mol) at 0–10 °C for 2 h. The formed precipitate was collected by filtration, washed with diethyl ether, dried, and crystallized from a mixture of ethanol–water (1:1). Physicochemical characterizations of potassium *N*-acylhydrazinecarbodithioates were reported elsewhere^31,76–78^.

### General procedure for the synthesis of s-triazoles (2)

Corresponding potassium *N*-acylhydrazinecarbodithioates (0.01 mol) were treated with hydrazine hydrate (0.015 mol) in water (5 mL), and the mixture was heated under reflux for 5 h. Water (10 mL) was added, and the pH was adjusted to 2–3 with 3M HCl. The formed precipitate was collected by filtration, washed with water, dried, and crystallized from ethanol. Physicochemical characterizations of **2a**, **2c–2e** (Table 4) were reported elsewhere^78–81^.

4-amino-3-(naphtalen-1-yl)-4,5-dihydro-1H-1,2,4-triazole-5-thione (**2d**) Yield: 91%, m.p. 263–5 °C, ^1^H NMR (300 MHz): 4.50 (s, 2H, NH2), 7.83–7.97 (m, 2H, Ar–H), 8.15–8.31 (m, 2H, Ar–H), 8.68 (s, 1H, Ar–H), 9.50 (s, 1H, Ar–H), 14.19 (s, 1H, NH). Anal. calc. for C_11_H_9_N_5_S (%):C 54.31, H 3.73, N 28.79. Found: C 54.35, H 3.69, N 28.70.

### Docking methodology

Docking was performed using the FlexX, as implemented in the LeadIT software package (LeadIT version 2.3.2; BioSolveIT GmbH, Sankt Augustin, Germany, 2017). The crystal structure of sterol 14-alpha demethylase (CYP51) from Candida albicans in complex with the tetrazole-based antifungal drug candidate VT1161 (PDB id: 5TZ1) was downloaded from the Protein Data Bank (PDB). All steps of ligands and receptor preparation were carried out using default settings in BioSolveIT’s LeadIT software. Chain A was selected, and the binding site was defined to include residues within a 6.5 Å radius around the native ligand. Soft docking (allowing for a volume overlap of up to 100 Å^3^) was performed. The clash factor was set to 0.1. Other parameters were kept to default. The conformation with the most favorable binding score was then selected for a detailed evaluation of binding site interactions. For 2D visualization, the PoseView dock widget as implemented in LeadIT version 2.3.2 software was used. According to the PoseView, green curves represent hydrophobic contacts of at least 3 different ligand atoms to surrounding residues. H-bond intermolecular interactions are indicated by a purple dashed line. For 3D docking results visualization the Protein–Ligand Interaction Profiler (PLIP) web tool was used^82^.

### Antifungal activity assay

Antifungal susceptibility tests were performed using the broth microdilution assay according to the European Committee on Antimicrobial Susceptibility Testing (EUCAST; E. Def 9. 3. 2)^35^ guidelines. Briefly, stock solutions of the tested compounds were two-fold diluted with RPMI-1640 (with 4% glucose, l-glutamine, without bicarbonate, buffered at pH 7.0, Biowest) from 128 to 0.5 mg/L (final volume 100 µL) in flat bottomed, clear 96-wells plate. The final DMSO concentration did not exceed 1% v/v and did not influence the growth of microorganisms. Then, a volume of 100 µL of final working inoculum (2–5 × 10^5^ CFU/mL) was added to each well. Microtiter plates were incubated at 34 °C in a moist, dark chamber for 24 h. Endpoints were defined as the lowest concentration of the compound resulting in total inhibition (minimal inhibitory concentration 100%, MIC100) of visual growth compared to the growth in the control wells containing only quality control isolates (*T. rubrum* CBS 118892 and *M. canis* CBS 113480) and no tested agents. Amphotericin B, as well as ketoconazole (AK scientific), were used as control antifungal drugs. All evaluations were performed in triplicate. The fungicidal activity was evaluated by transferring of 50 µL aliquot from a plate’s well with MIC for a specific fungal strain to 2 mL of fresh RPMI-1640 medium. After 48 h incubation at 34 °C in a moist, dark chamber, the visual growth was determined by comparison to a clear medium.

### Antibacterial activity assay

The in vitro antibacterial activity of tested compounds was evaluated against a panel of reference strains of Gram-negative (*Escherichia coli* NCTC 8196, *Proteus vulgaris* ATCC 49990, *Proteus mirabilis* ATCC 29906, *Pseudomonas aeruginosa* NCTC 6749) and Gram-positive (*Staphylococcus aureus* ATCC 6538, *Staphylococcus aureus* ATCC 29213, *Staphylococcus epidermidis* ATCC 12228) bacterial species. Minimal inhibitory concentration (MIC) was determined as the lowest concentration of the compound preventing the growth of the tested microorganism using the microdilution method according to EUCAST guidelines (ISO 20776-1 (2006). The 96-well microplates were used; 50 µL aliquots of the recommended Mueller–Hinton broth with a series of twofold dilutions of the tested compound, in the range of final concentrations from 256 to 1 mg/L, were inoculated with 50 µL of standardized microbial suspensions (5 × 10^5^ CFU/mL). The final DMSO concentration did not exceed 1% v/v and did not influence the growth of microorganisms. The incubation was carried out at 37 °C for 18 h, and the optical density at 600 nm was measured. All evaluations were performed in triplicate.

### Cell viability assay

MTT mitochondrial activity assay (Sigma-Aldrich) was performed to measure cell viability. Murine fibroblasts, L929 cells (ATTC®-CCL-1, mouse fibroblasts) or human tumor HeLa cells (ATTC®-CCL-2™, human epithelial cells), were plated on 96-well, flat-bottomed plates at a density of 1 × 10^4^ cells/mL and cultivated in Iscove’s modified Dulbecco’s medium supplemented with 10% fetal bovine serum and penicillin/streptomycin (100 U/100 µg). The cell cultures were incubated at 37 °C in a humidified atmosphere with 10% CO_2_. After 18 h of incubation, the growth medium was removed, and 100 μL of medium supplemented with twofold dilutions of tested compounds in the concentration from 128 to 0.5 mg/L were added. The final concentration of DMSO did not exceed 1% and did not influence cell viability. After 24 h incubation, the medium was removed, and 0.5 mg/mL of MTT (3-(4,5-Dimethylthiazol-2-yl)-2,5-Diphenyltetrazolium Bromide) was added to each well, and the plates were incubated for the next 2 h at 37 °C, 10% CO_2_. Then, formazan crystals were solubilized in 150 μL DMSO. The optical density was measured at 570 nm. The results of the experiments were shown as mean arithmetic values of absorbance from 3 repeats in each of two independent experiments, and the percentage of viability inhibition in comparison to untreated control was calculated for each concentration of the tested compounds, and IC_50_ and IC_30_ values were determined^83–85^. Moreover, the selectivity index (SI) was calculated for each compound using the formula: SI = (IC_50_ for cell line L929/HeLa)/(geometric mean (GM) MIC values for fungal stains).

### Cell viability determination by resazurin reduction assay (RRA)

Resazurin dye solution (ThermoFisher) was added to the germinated spores of *T. rubrum* (10% v/v) in 96-well plates to measure fungus viability. The plates were then incubated at 37 °C for 24 h. The absorbance at 570 nm with a reference filter 600 nm was registered in an automated plate reader (SpectraMax i3x, Molecular Devices). Cell viability was determined using the following equation:

$${\mathbf{Cell}} \;{\mathbf{viability}} \% = \frac{{{\text{Abs}}EXP}}{{{\text{Abs}}CT}}{ } \times { }100{\text{\% }}$$

where Abs*EXP* is the absorbance (difference between 570 and 600 nm) in the different concentrations of **2d** compound (128–0.5 mg/L) incubated with germinated spores of *T. rubrum,* and Abs*CT* is the absorbance in control cells (germinated spores of *T. rubrum* untreated with **2d** compound).

### White-light microscopy (WLM)

The effect of **2d** compound on the hyphal morphology of *T. rubrum* CBS was evaluated using white-light microscopy. The inoculum of germinated spores, 2–5 × 10^5^ CFU/mL, was transferred into liquid Sabouraud medium without **2d** compound (control) and the one containing **2d** compound at a concentration corresponding to 1 × MIC (32 mg/L). The observation using camera light microscopy (Nikon Eclipse E-2000, Nikon, Japan, with DeltaPix Camera) at a total magnification of × 400 (Scanning objective × 40 and Eyepiece × 10).

### Scanning electron microscopy (SEM)

To investigate the effect of **2d** compound on *T. rubrum* CBS cells, scanning electron microscopy was applied. The healthy volunteer nails were cut into small pieces of 2.5 × 3.5 mm^2^ and sterilized at 121 °C for 15 min. The sterilized nail fragments were exposed to a spore suspension containing 2–5 × 10^5^ CFU/mL for 2 h at 28 °C. The nails were then removed from the spore suspension and placed in a 24-well plate containing MM-Cove medium^86^. **2d** solution was added at concentrations 0.5 × MIC (16 mg/L), 1 × MIC (32 mg/L), and 2 × MIC (64 mg/L). After 24 h of exposure to **2d**, the nail fragments were fixed with 2.5% glutaraldehyde and dehydrated in a graded ethanol series. Then, the samples were placed into a critical point dryer (Leica EM CPD 3000) and were coated with ionized gold (Leica EM ACE 200). The nail fragments were observed in the Phenom ProX Scanning Electron Microscope belonging to the Department of Invertebrate Zoology and Hydrobiology, University of Lodz. The SEM method was carried out following relevant guidelines and regulations. The study was approved by the institutional ethics committee (the Local Ethics Committee in Lodz, Poland). Written informed consent was obtained from the volunteer.

### Transmission electron microscopy (TEM)

The 100 µL of final working inoculum of *T. rubrum* germinated spores (2–5 × 10^5^ CFU/mL) were incubated for 24 h at 28 °C with agitation in 900 µL RPMI 1640 medium containing 2% glucose, 32 mg/L of **2d** compound, and without **2d** as control. The samples were fixed in 2.5% glutaraldehyde in PBS buffer, pH 7.0, for 3 h at 0–4 °C. After 3-step washing in the same buffer (15 min washing, 2 min centrifugation, 1500 g), the obtained pellets were suspended in 2% agarose in PBS, then post-fixated with 1% osmium tetraoxide for 2 h at 4 °C. Samples were dehydrated in a graded ethanol series (10–50%), stained with 50% uranyl acetate (12 h), and dehydration was continued in higher ethanol concentrations (70–100%) and with propylene oxide. The samples were gradually saturated with a mixture of Epon-Spur resin^87^ and propylene oxide and finally embedded in Epon-Spur resin. The ultrathin sections (70 nm) were obtained with an ultramicrotome (Ultracut E, Reichert Young, Germany). The sections were placed on formvar-coated nickel grids, and after staining with a saturated solution of uranyl acetate and subsequently with lead citrate^88^, they were examined in a transmission electron microscope (JEM 1010, JEOL, Japan) at 80 kV. Mycelial ultrastructure was observed for at least four independent samples for each treatment. The TEM analysis was performed in the Laboratory of Microscopic Imaging and Specialized Biological Techniques, Faculty of Biology and Environmental Protection, University of Lodz, Poland.

### RNA-seq analysis

The effect of the **2d** compound on the transcriptome of *T. rubrum* CBS was evaluated using RNA sequencing analysis. The germinated spores, 2–5 × 10^5^ CFU/mL, were initially inoculated into 50 mL of Sabouraud medium and incubated at 28 °C for 96 h under agitation (stationary growth phase). Next, the obtained mycelia were transferred into RPMI 1640 medium without 2d compound (control) and containing 2d compound at a concentration corresponding to 1 × MIC (32 mg/L), incubated at 28 °C for 24 h, and forced to adapt to a stress-inducing environment^53^. Total RNA was extracted from *T. rubrum* cells using RNeasy Plant Mini Kit (Qiagen) according to the manufacturer’s instructions, with the addition of DNase I to eliminate potential DNA contamination. Quantity and purity of the RNA were assessed using NanoPhotometerTM Pearl Version 1.0 (IMPLEN) and verified using an Agilent 2100 Bioanalyzer (Agilent, USA). Four independent RNA biological replicates were used for cDNA synthesis with TruSeq RNA library Kit (Illumina, USA), according to the manufacturers’ guidelines. The BioBank Lab, University of Lodz, Poland, constructed the libraries and performed the sequencing reactions on the Illumina NextSeq system (Illumina, USA). The reads were aligned with the Botwie2 algorithm^45^ against the *T. rubrum* genome downloaded from the Broad Institute’s Dermatophyte Comparative Database (ftp://ftp.broadinstitute.org/pub/annotation/fungi). Significance analysis by Student's t-test and fold-change in the expression of genes between **2d** compound and control were used to identify DEGs. The Benjamini and Hochberg method^89^ was used to calculate the adjusted P-values. The criterion of statistical significance was FDR < 0.05 (False Discovery Rates) and Log_2_FoldChange > 1.5. Genes were functionally categorized with the Gene Ontology (GO)terms assigned by the Blast2GO algorithm^90^.

### RT-qPCR validation

cDNA was synthesized using 2 μg of total RNA (used for RNA-seq analysis), RevertAid reverse transcriptase (Thermo Scientific, Waltham, MA, USA), and Random Hexamer Primers (5’-NNNNNN-3’; N = G, A, T or C) (Thermo Scientific, Waltham, MA, USA) following the manufacturer’s protocol. The qRT-PCR assay was conducted on a Rotor-Gene Q System (Qiagen, Hilden Germany) using SsoAdvanced Universal SYBR Green Supermix (2x) (Bio-Rad, Hercules, California, USA). Primers used are listed in Supplementary Table S5. The mixtures were subjected to an initial step at 95 °C for 1 min, followed by 40 cycles of denaturation at 95 °C for 15, annealing and polymerization at 60 °C for 1 min. Melting curve analysis was performed by heating the amplicon from 72 to 95 °C. Relative gene expression levels were calculated according to the 2^−ΔΔCT^ method, with *sdha* and *rpl*2 for *T. rubrum* (Supplementary Table S5) as the reference genes according to the MIQE (Minimum Information for Publication of Quantitative Real-Time PCR Experiments) guidelines^91^. Statistical analysis was performed using one-way ANOVA test using GraphPad Prism version 7.00 for Windows (GraphPad Software, La Jolla, California, USA).

### Institutional review board statement

The use of human nails in experimental protocol was approved by the Local Ethics Committee in Lodz (Poland), protocol code: 9/KBBN-UŁ/II/2019, date of approval: 08/04/2019.

### Supplementary Information

Supplementary Figure S1.

Supplementary Figure S2.

Supplementary Figure S3.

Supplementary Figure S4.

Supplementary Table S1.

Supplementary Table S2.

Supplementary Table S3.

Supplementary Table S4.

Supplementary Table S5.

Supplementary Table S6.

Supplementary Table S7.

## Data Availability

All transcriptomic data are available at NCBI site under the BioProject PRJNA723549 (https://www.ncbi.nlm.nih.gov/bioproject/PRJNA723549).
