# Supplementary material for: Evaluation of the antidermatophytic activity of potassium salts of N-acylhydrazinecarbodithioates and their aminotriazole-thione derivatives
Source: Sci Rep. 2024 Feb 12;14:3521. doi: 10.1038/s41598-024-54025-9 (PMC10861498; doi:10.1038/s41598-024-54025-9)
Supplement: Supplementary file 1 — Supplementary Figure S1. [file 41598_2024_54025_MOESM1_ESM.pdf]

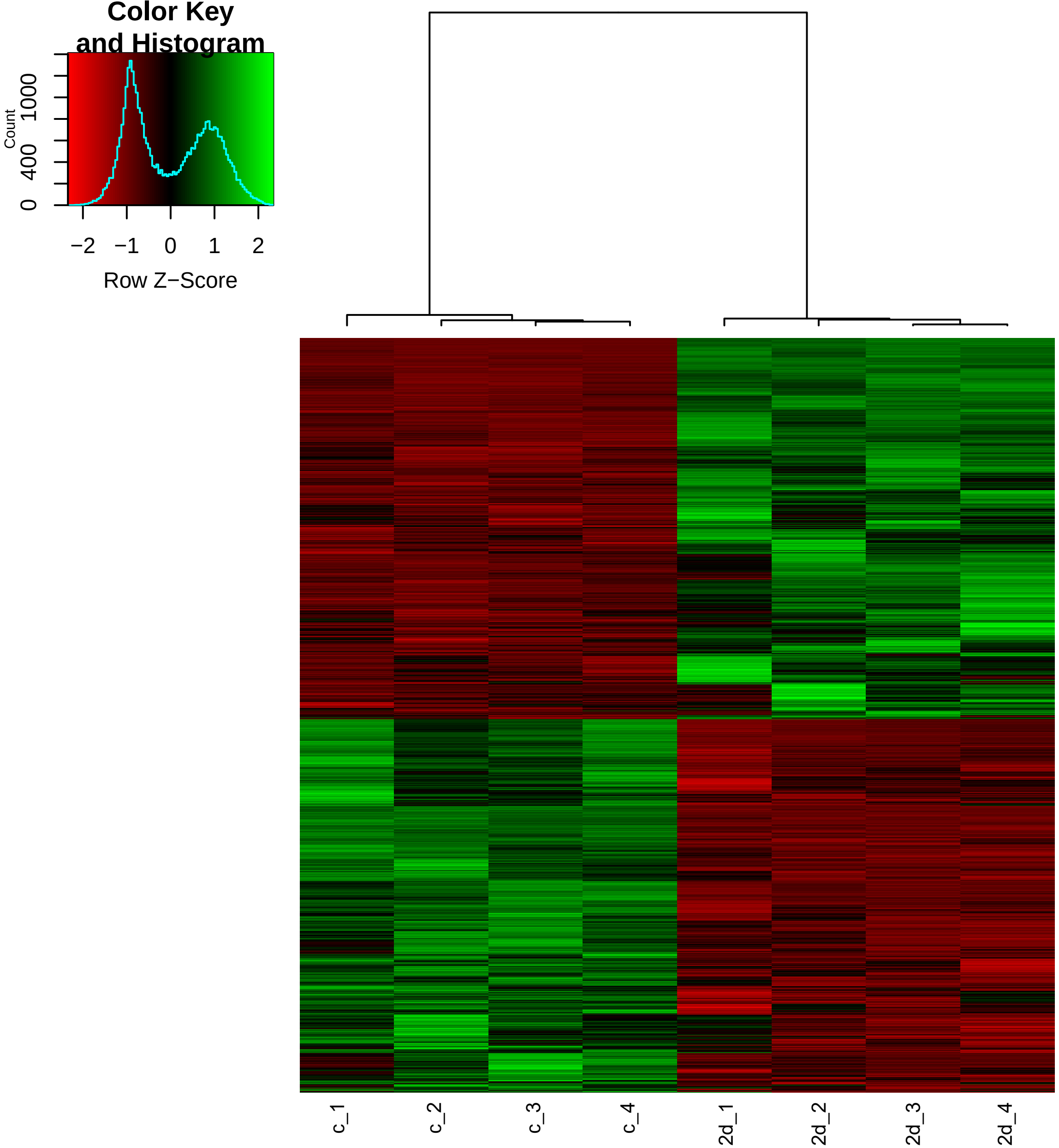

Fig. S1. RNA-seq analysis of *T. rubrum* CBS 120358 after exposure to the 2d compound. Heat map hierarchical clustering indicates differentially expressed genes between 2d compound samples and control samples. Quadruple biological replicates were performed for each group.
