# Supplementary material for: Evaluation of the antidermatophytic activity of potassium salts of N-acylhydrazinecarbodithioates and their aminotriazole-thione derivatives
Source: Sci Rep. 2024 Feb 12;14:3521. doi: 10.1038/s41598-024-54025-9 (PMC10861498; doi:10.1038/s41598-024-54025-9)
Supplement: Supplementary file 2 — Supplementary Figure S2. [file 41598_2024_54025_MOESM2_ESM.pdf]

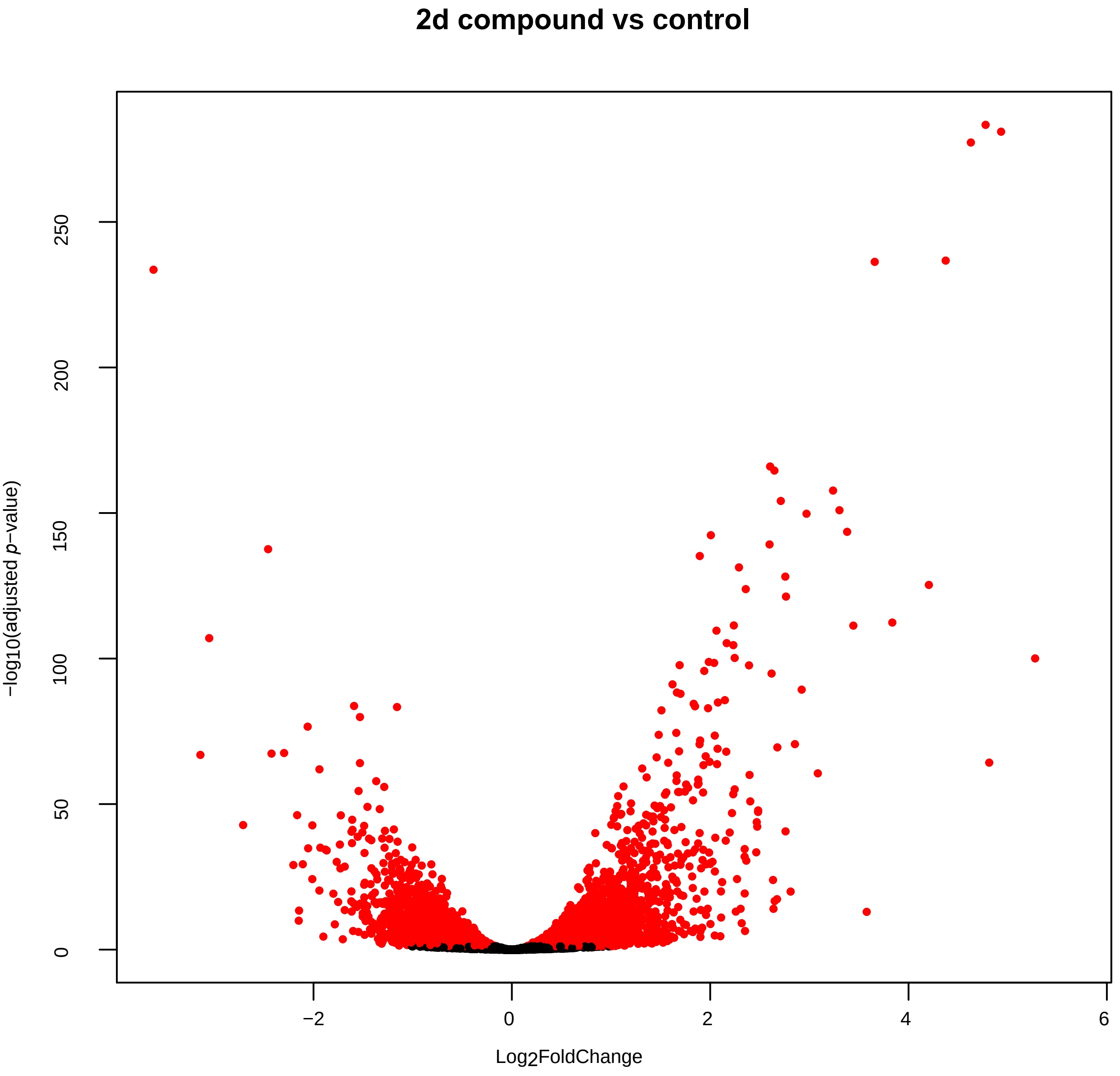

Fig. S2. A volcano plot of differentially expressed genes (DEGs). Y-axis denotes − log10-P- value while X-axis shows log2FoldChange values. The scattered dots represent each gene, the grey dots indicate genes with no significant differences, and the red dots indicate up-regulated and down-regulated genes with significant differences.
