# Supplementary material for: Evaluation of the antidermatophytic activity of potassium salts of N-acylhydrazinecarbodithioates and their aminotriazole-thione derivatives
Source: Sci Rep. 2024 Feb 12;14:3521. doi: 10.1038/s41598-024-54025-9 (PMC10861498; doi:10.1038/s41598-024-54025-9)
Supplement: Supplementary file 3 — Supplementary Figure S3. [file 41598_2024_54025_MOESM3_ESM.pdf]

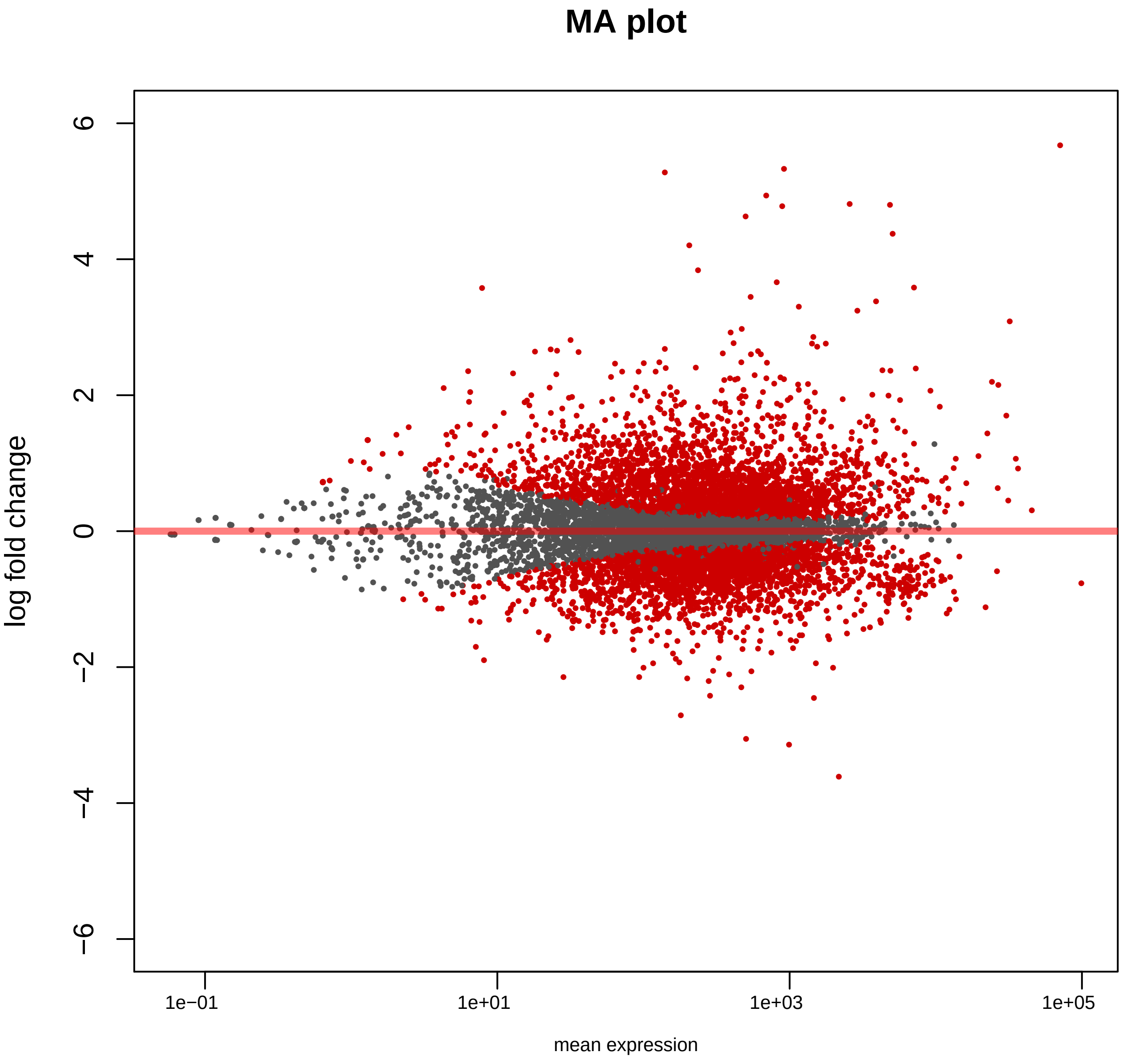

Fig. S3. MA plot shows the mean of the normalized counts versus the log2FoldChange for all differentially expressed genes (DEGs) in *T. rubrum* CBS 120 358 after exposure to the 2d compound
