# Supplementary material for: Evaluation of the antidermatophytic activity of potassium salts of N-acylhydrazinecarbodithioates and their aminotriazole-thione derivatives
Source: Sci Rep. 2024 Feb 12;14:3521. doi: 10.1038/s41598-024-54025-9 (PMC10861498; doi:10.1038/s41598-024-54025-9)
Supplement: Supplementary file 4 — Supplementary Figure S4. [file 41598_2024_54025_MOESM4_ESM.pdf]

A

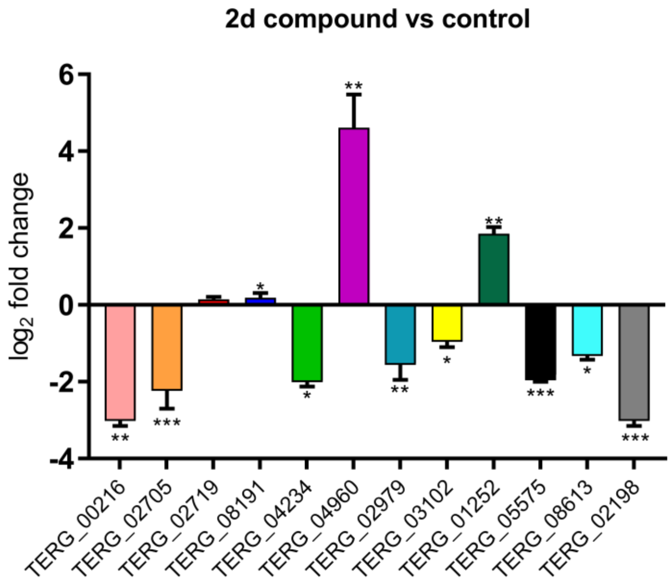

B

Accession no.	Gene Product Name	2 d compound vs control	
		**RNA-seq**	**qPCR**
TERG_00216	endochitinase	-1.18	-2.97
TERG_02705	class III chitinase	-	-2.19
TERG_02719	glycosyl hydrolase	0.78	0.14
TERG_08191	glucooligosaccharide oxidase	-0.48	0.19
TERG_04234	hydrophobin	-0.77	-1.96
TERG_04960	glutathione transferase	1.41	4.61
TERG_02979	Delta (24(24(1))-sterol reductase (erg4)	-0.55	-1.51
TERG_03102	sterol-24-C-methyltransferase (erg6)	-0.46	-0.91
TERG_01252	catalase A	0.72	1.83
TERG_05575	MFS multidrug transporter	-0.66	-1.81
TERG_08613	multidrug resistance protein (TruMDR2)	-0.76	-1.28
TERG_02198	CamK protein kinase	-0.60	-2.97

Fig. S4. Validation of differentially expressed genes using qRT-PCR. (A) Gene expression levels are represented as log_2FoldChange relative to the control condition (24 h – 2d compound x 24 h – control). The asterisk indicates the statistical significance as determined using ANOVA followed by Tukey's test (*P<0.05; **P<0.01, ***P<0.001). (B) Comparison of gene expression levels determined by RNA-seq with those evaluated by qRT-PCR.
