# Supplementary material for: Evaluation of the antidermatophytic activity of potassium salts of N-acylhydrazinecarbodithioates and their aminotriazole-thione derivatives
Source: Sci Rep. 2024 Feb 12;14:3521. doi: 10.1038/s41598-024-54025-9 (PMC10861498; doi:10.1038/s41598-024-54025-9)
Supplement: Supplementary file 5 — Supplementary Table S1. [file 41598_2024_54025_MOESM5_ESM.pdf]

TABLE S1 The fungicidal activity of **1a-e, 2a-e** expressed as growth inhibition obtained at a minimal inhibitory concentration (MIC). The symbol "+" signifies the presence of visible growth, while the symbol "-" indicates complete inhibition of growth. For strains that were not sensitive to compounds (MIC >128 mg/L) the fungicidal activity was not determined (nd).

Fungal Growth									
	**1a**	**2a**	**1b**	**1c**	**2c**	**1d**	**2d**	**1e**	**2e**
Trichophyton rubrum CBS	+	+	+	+	-	+	-	nd	nd
Trichophyton rubrum 127/07	nd	-	nd	+	-	+	-	nd	nd
Trichophyton rubrum 144/10	+	-	nd	+	-	+	-	nd	nd
Trichophyton rubrum 451/04	+	+	nd	nd	-	nd	-	nd	nd
Trichophyton interdigitale CBS	nd	-	nd	+	-	+	-	+	+
Trichophyton interdigitale 445/10	+	-	+	+	-	+	-	nd	+
Trichophyton granulosum 49/10	nd	+	nd	+	+	+	+	+	+
Trichophyton granulosum 175/07	nd	nd	nd	nd	+	+	+	nd	nd
Trichophyton tonsurans CBS	nd	-	nd	+	-	nd	-	nd	nd
Trichophyton tonsurans 170/08	nd	+	nd	+	+	nd	+	nd	nd
Chrysosporium keratinophilum CBS	nd	nd	nd	nd	nd	nd	+	nd	nd
Microsporum canis CBS	nd	+	+	+	+	+	+	+	+
Microsporum canis 31	nd	+	+	+	+	+	+	+	+
Microsporum canis 150	+	+	+	+	+	nd	+	+	+
