# Supplementary material for: Evaluation of the antidermatophytic activity of potassium salts of N-acylhydrazinecarbodithioates and their aminotriazole-thione derivatives
Source: Sci Rep. 2024 Feb 12;14:3521. doi: 10.1038/s41598-024-54025-9 (PMC10861498; doi:10.1038/s41598-024-54025-9)
Supplement: Supplementary file 6 — Supplementary Table S2. [file 41598_2024_54025_MOESM6_ESM.pdf]

TABLE S2 Antibacterial activity of **1a-e, 2a-e** expressed as the minimal inhibitory concentration resulted in 30% (MIC_{30}), 50% (MIC_{50}), and 70% (MIC_{70}) bacteria growth inhibition [mg/L]

Strain	***E. coli*** **NCTC 8196**	***S. aureus*** **ATCC 6538**	***S. aureus*** **ATCC 29213**	***S. epidermidis*** **ATCC 12228**	***E. faecalis*** **ATCC 29213**	***P. vulgaris*** **ATCC 49990**	***P. mirabilis*** **ATCC 29906**	***P. aeruginosa*** **NCTC 6749**
1a								
**MIC_{50}**	>256	>256	>256	>256	>256	>256	>256	>256
**MIC_{70}**	>256	>256	>256	>256	>256	>256	>256	>256
2a								
**MIC_{50}**	>256	>256	>256	>256	>256	>256	>256	>256
**MIC_{70}**	>256	>256	>256	>256	>256	>256	>256	>256
1b								
**MIC_{50}**	64	128	64	16	>256	128	64	>256
**MIC_{70}**	256	>256	256	>256	>256	>256	128	>256
1c								
**MIC_{50}**	>256	>256	>256	>256	>256	>256	>256	>256
**MIC_{70}**	>256	>256	>256	>256	>256	>256	>256	>256
2c								
**MIC_{50}**	>256	>256	>256	>256	>256	>256	>256	>256
**MIC_{70}**	>256	>256	>256	>256	>256	>256	>256	>256
1d								
**MIC_{50}**	>256	>256	>256	>256	>256	>256	>256	>256
**MIC_{70}**	>256	>256	>256	>256	>256	>256	>256	>256
2d								
**MIC_{50}**	32	256	>256	>256	>256	>256	>256	>256
**MIC_{70}**	256	>256	>256	>256	>256	>256	>256	>256
1e								
**MIC_{50}**	>256	>256	>256	>256	>256	>256	>256	>256
**MIC_{70}**	>256	>256	>256	>256	>256	>256	>256	>256
2e								
**MIC_{50}**	>256	>256	>256	>256	>256	>256	>256	>256
**MIC_{70}**	>256	>256	>256	>256	>256	>256	>256	>256
