# Supplementary material for: Evaluation of the antidermatophytic activity of potassium salts of N-acylhydrazinecarbodithioates and their aminotriazole-thione derivatives
Source: Sci Rep. 2024 Feb 12;14:3521. doi: 10.1038/s41598-024-54025-9 (PMC10861498; doi:10.1038/s41598-024-54025-9)
Supplement: Supplementary file 7 — Supplementary Table S3. [file 41598_2024_54025_MOESM7_ESM.pdf]

Table S3 Comparison of selectivity index (SI) values of the tested compounds **1a-1e**, **2a**–**2d** and antifungal agents amphotericin B and ketoconazole. The SI was calculated for each compound using the formula: SI = (IC_{50} for cell line L929/HeLa)/MIC values for fungal stains). A favorable SI > 1.0 indicates a compound with efficacy against fungal strains greater than the toxicity against mammalian cells. MIC and IC_{50} values are presented in mg/L.

**Selectivity Index (SI)**											
	**1a**	**2a**	**1b**	**1c**	**2c**	**1d**	**2d**	**1e**	**2d**	**A**	**K**
**MIC range**	128->128	16-128	32-128	64-128	32-128	64-128	32-128	64-128	16-128	4-16	0.5-4
**L929 IC_{50}**	>128	>128	>128	>128	>128	>128	>128	>128	>128	64	100
**HeLa IC_{50}**	>128	>128	>128	>128	>128	>128	>128	>128	>128	>128	128
**SI**	≥1	>1-8	>1-4	>1-2	>1-4	>1-2	>1-4	>1-2	>1-8	4- >32	25-256

A - Amphotericin B, **K** – Ketoconazole
