# Supplementary material for: Evaluation of the antidermatophytic activity of potassium salts of N-acylhydrazinecarbodithioates and their aminotriazole-thione derivatives
Source: Sci Rep. 2024 Feb 12;14:3521. doi: 10.1038/s41598-024-54025-9 (PMC10861498; doi:10.1038/s41598-024-54025-9)
Supplement: Supplementary file 9 — Supplementary Table S5. [file 41598_2024_54025_MOESM9_ESM.pdf]

TABLE S5 List of target genes and primer sequences for qRT-PCR

Accession no.	Gene product name	Primers (5’-3’)	Amplicon (bp)	Efficiency (%)	References
TERG_00216	endochitinase	**F**: TCGACAACGGTCTAGGGAGGT<br>**R**: CCTCGACGAAGGTAGCAATC	102	98.21	[53]
TERG_02705	class III chitinase	**F**: CAAGGAGATGCTGGAAGGAG<br>**R**: AGCCGAGGACGAAGTTGTTA	96	101.27	[53]
TERG_02719	glycosyl hydrolase	**F**: AGCCCTTCTTCCTCATCCTC<br>**R**: GGTCGGGTAGAACTTGTCCA	101	96.99	[53]
TERG_08191	glucooligosaccharide oxidase	**F**: CAGCTCAATGCTTTTGGAGAG<br>**R**: TCTCCGTCTACCATGGACTGT	92	100.79	[53]
TERG_04234	hydrophobin	**F**: GGCATACATCTTGGTGGTTTC<br>**R**: CAGACAGTGGAGGTGGATGTT	95	109.12	[53]
TERG_04960	glutathione transferase	**F**: CCCAAACACCGACCTGAA<br>**R**: TCGTTGGTATCGTGTGGAAAG	105	97.01	[47]
TERG_02979	Delta (24(24(1))-sterol reductase (*erg4*)	**F**: CCTTCCCTTGGTTTTACTTCG<br>**R**: TGTAGGGAACAGCCTTCTCG	126	100.80	[48]
TERG_03102	sterol-24-C-methyltransferase (*erg6*)	**F**: GGCTGACAAAGGGGACATAA<br>**R**: TGGTCACTCGAAGAGCACTG	98	99.14	[48]
TERG_01252	catalase A	**F**: CCACTGGTGATGCAGTCAAT<br>**R**: CACACCATAGCTCTCCACGA	107	102.11	[48]
TERG_05575	MFS multidrug transporter	**F**: GAGGTTTGTCTGGATCGTCTG<br>**R**: CCCTGATTCAAGTCATACGAG	87	92.11	[47]
TERG_08613	multidrug resistance protein (*Tru*MDR2)	**F**: TGACGAGGCTACATCAGCAC<br>**R**: GTCGGTGAGCAACAGCAATA	104	97.80	[48]
TERG_02198	CamK protein kinase	**F**: CAAGGAATCCCAGAGCAAAG<br>**R**: GTAGAATTCGCACCGAAAGC	112	103.49	[48]
TERG_02073	*sdha* (reference gene)	**F:** CTGTTGACTTGCGCTACC<br>**R:** GGAAGGTAAGAGTGTGCTTC	127	101	[77]
TERG_04033	*rpl2* (reference gene)	**F:** GGATCTATATTCACGGCTCG<br>**R:** TGGATGATGTTCTTCACGAC	113	102	[77]
