# Supplementary material for: Evaluation of the antidermatophytic activity of potassium salts of N-acylhydrazinecarbodithioates and their aminotriazole-thione derivatives
Source: Sci Rep. 2024 Feb 12;14:3521. doi: 10.1038/s41598-024-54025-9 (PMC10861498; doi:10.1038/s41598-024-54025-9)
Supplement: Supplementary file 10 — Supplementary Table S6. [file 41598_2024_54025_MOESM10_ESM.pdf]

TABLE S6 RNA-seq results for genes related to the membrane transport and cell wall integrity modulated in response to the **2d** compound exposure

ID	Gene Product Name	2 d compound vs control (24 h)
**GENES RELATED TO THE TRANSPORT**		
TERG_08591	ABC transporter	0.61
TERG_03878	ABC fatty transporter	0.68
TERG_00286	ABC transporter	- 0.57
TERG_00955	ABC efflux transporter	0.90
TERG_01443	ABC multidrug transporter	- 0.66
TERG_05776	peroxisomal ABC transporter	0.80
TERG_06419	ABC transporter	1.14
TERG_01489	MFS transporter	3.11
TERG_01623	MFS transporter	0.30
TERG_01634	MFS transporter	-0.36
TERG_01655	MFS monocarboxylate transporter	-0.44
TERG_01820	MFS drug transporter	-1.50
TERG_02369	MFS transporter	-0.43
TERG_02543	MFS transporter	0.93
TERG_02545	MFS monocarboxylate transporter	1.43
TERG_04094	MFS transporter	-0.46
TERG_04182	MFS sugar transporter	0.45
TERG_04400	MFS monosaccharide transporter	1.33
TERG_04711	MFS drug transporter	-0.75
TERG_05055	MFS transporter	-0.76
TERG_05153	MFS transporter	0.58
TERG_05199	MFS multidrug transporter	1.58
TERG_05258	MFS transporter	1.36
TERG_05429	MFS transporter	1.67
TERG_05466	MFS transporter	-0.68
TERG_05526	MFS multidrug transporter	0.84
TERG_05895	MFS transporter	0.72
TERG_07884	MFS multidrug transporter	1.29
TERG_08336	MFS multidrug transporter	0.97
TERG_11599	MFS transporter	0.88
TERG_05575	MFS multidrug transporter	-0.66
TERG_12579	MFS transporter	-0.63
TERG_00706	MFS transporter	-1.21
TERG_00776	MFS transporter	0.76
TERG_00820	MFS multidrug resistance transporter	1.26
TERG_03240	transmembrane efflux protein	0.65
TERG_08613	multidrug resistance protein (*TruMdr*2)	-0.76
**GENES RELATED TO THE CELL WALL**		
TERG_00060	GPI anchored cell wall protein	4.47
TERG_00216	endochitinase	-1.18
TERG_00342	phosphoglucomutase	0.44
TERG_00625	glycolipid anchored Surface protein (GAS1)	0.52
TERG_00638	cellobiose dehydrogenase	-1.35
TERG_00707	GPI anchored serine-threonine rich protein	-0.50
TERG_01127	1,3-beta-glucan synthase component FKS1	0.63
TERG_01967	GPI anchored protein	-0.94

TERG_02517	N-acetyltransferase, GNAT family	-1.46
TERG_02719	glycosyl hydrolase	0.78
TERG_02742	glycosyl hydrolase	-0.60
TERG_03144	glucose-6-phosphate isomerase	0.60
TERG_03223	N-acetylglucosamine-6-phosphate deacetylase	-1.21
TERG_03353	endoglucanase	0.83
TERG_03618	class V chitinase	0.84
TERG_03624	SUN domain protein (Uth1)	0.63
TERG_03896	mannose-6-phosphate isomerase	0.83
TERG_04234	hydrophobin	-0.77
TERG_04564	mixed-linked glucanase	-0.89
TERG_05618	Lcc2	0.97
TERG_05626	chitinase	0.42
TERG_06016	glycosyl hydrolase	0.36
TERG_06144	cell wall serine-threonine-rich galactomannoprotein Mp1	0.52
TERG_06242	glucanase	-0.69
TERG_06397	alpha-1,2-mannosyltransferase	-0.64
TERG_06638	endochitinase	1.30
TERG_06749	cell wall protein	0.66
TERG_06929	chitinase	0.47
TERG_07406	alpha-mannosidase	-2.42
TERG_07408	acetyl transferase, GNAT family	-1.09
TERG_07456	cell wall protein PhiA	1.13
TERG_07597	SOK1	-1.48
TERG_07641	GPI-anchored cel wall protein Pst1	0.45
TERG_07657	chitin synthase class VI	0.57
TERG_07662	Mannosylphosphate tranferase	-0.50
TERG_07734	O-methyltransferase	-0.65
TERG_07987	GNAT family acetyltransferase	-0.46
TERG_08191	glucoologosaccharide oxidase	-0.48
TERG_08286	GPI anchored CFEM domain protein	1.07
TERG_08498	triosephosphate isomerase	0.83
TERG_08969	cytosolic Cu/Zn superoxide dismutase	1.58
TERG_11951	Prp 6 CRoW domain-containing protein	1.49
TERG_12107	1,4-alpha-glucan-branching enzyme	-0.61
TERG_12281	glycosyl hydrolase	0.76
TERG_12282	glycosyl hydrolase	1.03
