# Supplementary material for: Evaluation of the antidermatophytic activity of potassium salts of N-acylhydrazinecarbodithioates and their aminotriazole-thione derivatives
Source: Sci Rep. 2024 Feb 12;14:3521. doi: 10.1038/s41598-024-54025-9 (PMC10861498; doi:10.1038/s41598-024-54025-9)
Supplement: Supplementary file 11 — Supplementary Table S7. [file 41598_2024_54025_MOESM11_ESM.pdf]

| potassium N-acylhydrazinecarbodithioates | | s-triazoles | |
|---|---|---|---|
| **1a** | 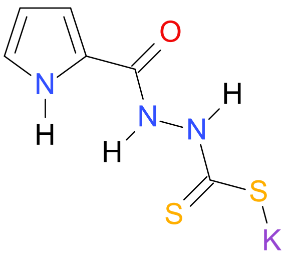<br>References:<br><br>Siwek A, Plech T, Trotsko N, Kosikowska U, Malm A, Dzitko K, Paneth P. Conformational preference of potassium salts of N-acylhydrazinecarbodithioates with antifungal activity. Combined experimental and theoretical approach. Current Computer-Aided Drug Design 2014, 10 (3): 205-216. doi: 10.2174/1573409910666140521152701 | **2a** | 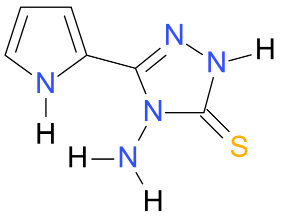<br>References:<br><br>Cai, S.X.; Drewe, J.A.; Zhang, H.Z.; Kasibhatla, S.; Claassen, G.; Sirisoma, N.S.; Kemnitzer, W.E. Preparation of 3,6-diaryl-7H [1,2,4] triazolo [3,4b][1,3,4] thiadiazines and analogs as activators of caspases and inducers of apoptosis. PCT Int. Appl. 2008, WO 2008011045 A2 20080124. |
| **1b** | 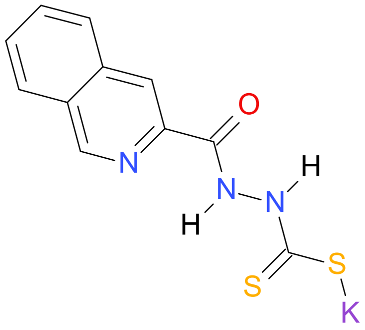<br>References:<br><br>Siwek A, Plech T, Trotsko N, Kosikowska U, Malm A, Dzitko K, Paneth P. Conformational preference of potassium salts of N-acylhydrazinecarbodithioates with antifungal activity. Combined experimental and theoretical approach. Current Computer-Aided Drug Design 2014, 10 (3): 205-216. doi: 10.2174/1573409910666140521152701 | **2b** | 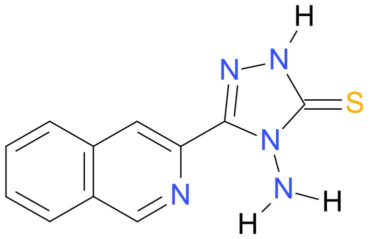<br>References:<br><br>Siwek A, Plech T, Trotsko N, Kosikowska U, Malm A, Dzitko K, Paneth P. Conformational preference of potassium salts of N-acylhydrazinecarbodithioates with antifungal activity. Combined experimental and theoretical approach. Current Computer-Aided Drug Design 2014, 10 (3): 205-216. doi: 10.2174/1573409910666140521152701 |

| | | | |
|---|---|---|---|
| **1c** | 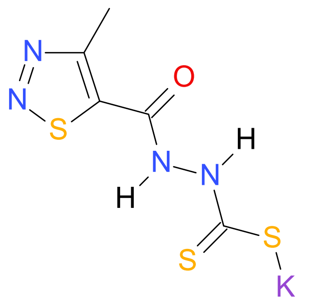<br><br>References:<br><br>Siwek A, Plech T, Trotsko N, Kosikowska U, Malm A, Dzitko K, Paneth P. Conformational preference of potassium salts of N-acylhydrazinecarbodithioates with antifungal activity. Combined experimental and theoretical approach. Current Computer-Aided Drug Design 2014, 10 (3): 205-216. doi: 10.2174/1573409910666140521152701 | **2c** | 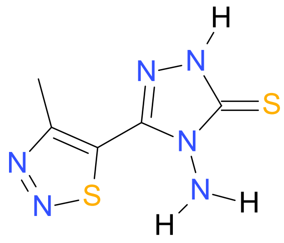<br><br>**CAS:** [64369-18-2]<br><br>References:<br><br>Shafiee, A.; Lalezari, I.; Mirrashed, M.; Nercesian, D. 1,2,3-Selenadiazolyl-1,3,4-oxadiazole, 1,2,3-thiadiazolyl-1,3,4-oxadiazole and 5-(1,2,3-thiadiazolyl)-s-triazolo[3,4,-b]-1,3,4-thiadiazoles. Journal of Heterocyclic Chemistry 1977, 14 (4): 567-71. doi: 10.1002/jhet.5570140407 |
| **1d** | 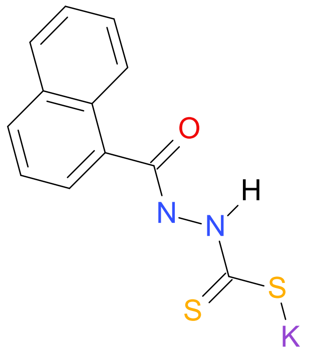<br><br>References:<br><br>Siwek A, Plech T, Trotsko N, Kosikowska U, Malm A, Dzitko K, Paneth P. Conformational preference of potassium salts of N-acylhydrazinecarbodithioates with antifungal activity. Combined experimental and theoretical approach. Current Computer-Aided Drug Design 2014, 10 (3): 205-216. doi: 10.2174/1573409910666140521152701 | **2d** | 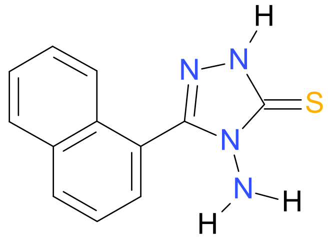<br><br>**CAS:** [127227-32-1]<br><br>References<br><br>Mohan, Jag; Anjaneyulu, G. S. R.; Yamini, K. V. S. Heterocyclic systems containing bridgehead nitrogen atom: synthesis and antimicrobial activity of s-triazolo[3,4-b][1,3,4]thiadiazoles, s-triazolo[3,4-b][1,3,4] thiadiazines and s triazolo[3',4':2,3][1,3,4] thiadiazino[5,6b]quinoxaline. Journal of the Indian Chemical Society 1991, 68 (8): 474-6. |

| | | | |
|---|---|---|---|
| **1e** | 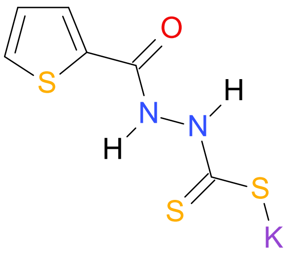<br><br>References:<br><br>Siwek A, Plech T, Trotsko N, Kosikowska U, Malm A, Dzitko K, Paneth P. Conformational preference of potassium salts of N-acylhydrazinecarbodithioates with antifungal activity. Combined experimental and theoretical approach. Current Computer-Aided Drug Design 2014, 10 (3): 205-216. doi: 10.2174/1573409910666140521152701 | **2e** | 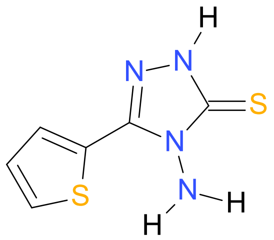<br><br>**CAS:** **61019-27-0**<br><br>References<br><br>Reid, Jack R.; Heindel, Ned D**.** Improved syntheses of 5-substituted-4-amino-3-mercapto-(4H)-1,2,4-triazoles**.** Journal of Heterocyclic Chemistry 1976, 13 (4): 925-6. doi: 10.1002/jhet.5570130450 |
